# Genetic basis for virulence differences of various *Cryptosporidium parvum* carcinogenic isolates

**DOI:** 10.1038/s41598-020-64370-0

**Published:** 2020-04-30

**Authors:** Christophe Audebert, Franck Bonardi, Ségolène Caboche, Karine Guyot, Hélène Touzet, Sophie Merlin, Nausicaa Gantois, Colette Creusy, Dionigia Meloni, Anthony Mouray, Eric Viscogliosi, Gabriela Certad, Sadia Benamrouz-Vanneste, Magali Chabé

**Affiliations:** 1 Gènes Diffusion, 3595, route de Tournai, 59501 Douai, France; 2 PEGASE-Biosciences, Institut Pasteur de Lille, Lille, France; 3 Bilille, Institut Pasteur de Lille, Lille, France; 4 Univ. Lille, CNRS, Inserm, CHU Lille, Institut Pasteur de Lille, U1019 – UMR 8204 – CIIL – Centre d’Infection et d’Immunité de Lille, Lille, France; 5 CNRS, Univ. Lille, Inria, UMR 9189 - CRIStAL - Centre de Recherche en Informatique Signal et Automatique de Lille, Lille, France; 6 Service d’Anatomie et de Cytologie Pathologiques, Groupement des Hôpitaux de l’Institut Catholique de Lille (GHICL), Lille, France; 7 Plateforme d’Expérimentations et de Hautes Technologies Animales, Institut Pasteur de Lille, Lille, France; 8 Délégation à la Recherche Clinique et à l’Innovation, Groupement des Hôpitaux de l’Institut Catholique de Lille, Lille, France; 9 Equipe Ecologie et biodiversité, Unité de Recherche Smart and Sustainable Cities, Faculté de Gestion Economie et Sciences, Institut Catholique de Lille, Lille, France

**Keywords:** Parasite biology, Parasite genomics

## Abstract

*Cryptosporidium parvum* is known to cause life-threatening diarrhea in immunocompromised hosts and was also reported to be capable of inducing digestive adenocarcinoma in a rodent model. Interestingly, three carcinogenic isolates of *C. parvum*, called DID, TUM1 and CHR, obtained from fecal samples of naturally infected animals or humans, showed higher virulence than the commercially available *C. parvum* IOWA isolate in our animal model in terms of clinical manifestations, mortality rate and time of onset of neoplastic lesions. In order to discover the potential genetic basis of the differential virulence observed between *C. parvum* isolates and to contribute to the understanding of *Cryptosporidium* virulence, entire genomes of the isolates DID, TUM1 and CHR were sequenced then compared to the *C. parvum* IOWA reference genome. 125 common SNVs corresponding to 90 CDSs were found in the *C. parvum* genome that could explain this differential virulence. In particular variants in several membrane and secreted proteins were identified. Besides the genes already known to be involved in parasite virulence, this study identified potential new virulence factors whose functional characterization can be achieved through CRISPR/Cas9 technology applied to this parasite.

## Introduction

*Cryptosporidium* apicomplexan parasites represent a major public health problem in humans and animals causing self-limited diarrhea in immunocompetent hosts and life-threatening disease in immunocompromised hosts, for which efficient drug therapy is still lacking. Particularly, the Global Enteric Multicenter Study (GEMS) revealed that *Cryptosporidium* was one of the four major pathogens responsible of moderate to severe cases of diarrhea among children in Africa and Asia^1^. The Global Burden of Disease 2015 Study also showed that *Cryptosporidium* was the second leading cause of death associated with diarrhea in children under 5 years of age^2^.

Currently, almost 40 *Cryptosporidium* species with a broad host range among vertebrates are recognized as valid, of which 20 species and genotypes have been identified in humans^3^. However, C. *hominis* and *C. parvum* are responsible for the majority of human infections^4^. Human is the major host for *C. hominis* while *C. parvum* is frequently reported both in humans and animals, particularly in bovids^4^.

Due mainly to the lack of continuous culture of the parasite, genomic studies of *Cryptosporidium* spp. took some time to be launched, compared to studies related to other apicomplexan parasites. The genomes of laboratory isolates of *C. parvum* IOWA^5^, *C. hominis* (TU502)^6^, and *C. muris* (RN66) (published in online public databases, e.g., CryptoDB http://cryptodb.org) were reported a decade ago. More recently, other genomes of *C. parvum* (including TU114 isolate)^7–9^ and *C. hominis* isolates (including UKH1 and UdeA01) were also available^9–11^. Genomes of additional *Crypstosporidium* species or genotypes such as *C. baileyi* TAMU- 09Q1 and *C. meleagridis* UKMEL1^12^, *C. andersoni*, *C. tyzzeri, C. ubiquitum* and *Cryptosporidium* chipmunk genotype 1^13^ have very recently been sequenced and released in CryptoDB. An improvement of the annotation of *C. parvum* IOWA genome^10^ and a recent annotation of *C. hominis* TU502_2012^12^ are now also available (CryptoDB).

The availability of sequence data for the entire genomes of *Cryptosporidium* spp. has contributed and will necessarily continue to contribute to the understanding of the fundamental biology of this parasite, but comparative genomics studies are still limited for this parasite^8,11,13,14^. In one of these comparative genomic studies, some multigene families that could explain differences in host specificity of *C. parvum* and *C. hominis* have been identified^11^. Moreover, comparison of *C. parvum* and *C. hominis* genomes showed that their chromosomes are completely syntenic and exhibit 95% to 97% of sequence similarity at the nucleotide level^10,14^. However, these two *Cryptosporidium* species possess many distinct phenotypic traits. It has therefore been assumed that phenotypic differences between these two species must be the result of slight sequence divergence, such as single nucleotide variants (SNVs) and/or small insertions/deletions (indels) as well as differences in gene regulation^14,15^.

Another comparative genomic study has suggested the potential role of genetic recombination in the emergence and evolution of virulent subtypes^11^. However, further studies are needed to fully understand the virulence of this parasite, and to identify for example, genetic determinants for virulence of various *Cryptosporidium* species and isolates. Until now, reports on characterization of *Cryptosporidium* virulence factors were scarce due to the fact that *in vitro* cultivation and transfection techniques with this parasite were difficult^15^. Only recently, the transfection of *C. parvum* sporozoites was reported using CRISPR/Cas9 technology^16^.

Interestingly, we formerly reported that *C. parvum* isolates of animal or human origin were able to induce digestive adenocarcinoma in a rodent model^17–21^. However, when we compared phenotypic differences between them, three carcinogenic *C. parvum* isolates named DID, TUM1 and CHR (Table 1) in our possession and isolated from fecal samples of naturally infected animals or humans, exhibited higher virulence than the commercially also carcinogenic *C. parvum* IOWA isolate, maintained by serial propagation in calf being its genome the reference genome for *C. parvum* (Table 1)^5^. Particularly, mice inoculated with the three more virulent isolates showed more severe clinical manifestations, higher mortality rate, and faster neoplastic lesion progression (Table 1) and only these mice developed extra gastro-intestinal lesions. Thus, in this work and in order to contribute to the understanding of *Cryptosporidium* virulence, whole genomes of these three highly virulent carcinogenic *C. parvum* isolates were sequenced and compared with that of the *C. parvum* IOWA isolate^5^. After DNA extraction of IMS (ImmunoMagnetic Separation)-purified parasites and Whole Genome Amplification (WGA), sequencing of Multiple Displacement Amplification (MDA) products was then performed using Ion Torrent sequencing technology for DID and TUM1 and Illumina technology for the more recently obtained *C. parvum* CHR isolate. In order to limit the amount of false positive mutations regularly observed in comparative genomic analyses, the analytical procedure consisted of using two different bioinformatics pipelines to determine the genetic determinants common to the three most virulent *C. parvum* isolates compared to the *C. parvum* IOWA reference genome. From our comparative analysis 125 common SNVs corresponding to 90 coding DNA sequences (CDS) in the *C. parvum* genome were identified that could explain this high virulence. Of interest, we identified variants in several membrane and secreted proteins. Some of these genes were already known to be involved in parasite virulence, but this study has identified new potential virulence factors whose functional characterization is now possible using gene editing technologies.

**Table 1:** Phenotypic features of *C. parvum* experimental infection observed in severe combined immunodeficiency (SCID) mice after inoculation with different isolates: IOWA, TUM1, DID and CHR.

	*C. parvum* (IOWA)	*C. parvum* (TUM1)	*C. parvum* (DID)	*C. parvum* (CHR)
Length of time between *Cryptosporidium* isolation and mice infection	<1 month	<1 month	<1 month	5 months
Oocyst viability	> 95%	> 95%	> 95%	> 95%
Infection dose	10^5^	10^5^	10^5^	5.10^3^
Log10 of oocysts/mg of feces	4.32^a^	6.53^b^	6.15^c^	NA
Clinical manifestations	Rare and late onset: spiky hair, lethargy, prostration	Frequent: bloody diarrhea, spiky hair, lethargy, prostration	Frequent: bloody diarrhea, spiky hair, lethargy, prostration and one case of rectal prolapse	Frequent: spiky hair, lethargy, prostration
Time of onset of digestive neoplastic lesion (Days Post-Infection, PI)	45	20	40^d^	15
The most severe observed neoplastic lesion grade^e^	5	4^f^	5	4^f^
Localization of the most severe lesion	Antropyloric region	Ileocaecal region	Ileocaecal region and biliary tree	Ileocaecal region
Extra gastro-intestinal lesions	No	Intraepithelial neoplasia in the biliary tree	Cholangiocarcinoma and vascular tumor emboli	Pancreatitis
Mortality rate at 60 days PI^g^	0% (0/80)	29% (7/24)	41% (10/24)	29% (4/14)
Maximum score of severity^h^	11	32.5	35	40

^a^Quantification before euthanasia at 57 days PI.

^b^Quantification before euthanasia at 35 days PI.

^c^Quantification before euthanasia at 60 days PI.

^d^For this animal experiment onset of neoplastic lesions before 40 days PI was not explored. This time corresponds to the first date of planned euthanasia. Thus, it is not excluded that the lesion appeared before.

^e^0, no lesion; 1, inflammation and/or regenerative changes; 2, low grade intraepithelial neoplasia (LGIEN); 3, High grade intraepithelial neoplasia (HGIEN); carcinoma *in situ* (limited to the epithelium) or intramucosal adenocarcinoma (invasion into the lamina propria through the basement membrane of glands); 4, submucosal adenocarcinoma when glands penetrate through the muscularis mucosae; 5, invasive adenocarcinoma with the invasion through the muscularis into the subserosa.

^f^This grade corresponds to the most severe lesion observed in mice that were alive at the time of euthanasia. It is not excluded that the ones who died before had a more severe lesion.

^g^This mortality concerned animals that died before planned euthanasia.

^h^The degree of severity of histological damage for each mouse was calculated by the sum of neoplastic lesions scores over five organs (stomach, liver, duodenum, ileocecal region and colon). In order to include the mortality as a factor of disease severity, each mouse which died before planned euthanasia was assigned a number of points equivalent to: 25 + ((60 – day of death)/2), where 25 corresponded to the maximum score of severity that can be reached by animals euthanized as planned, and 60 days corresponded to the end time of the experiment (slightly modified from Certad *et al*., 2010^18^).

NA: Not applicable

## Results

### **Phenotypic differences between the four*C. parvum*isolates**

**Figure 1:**
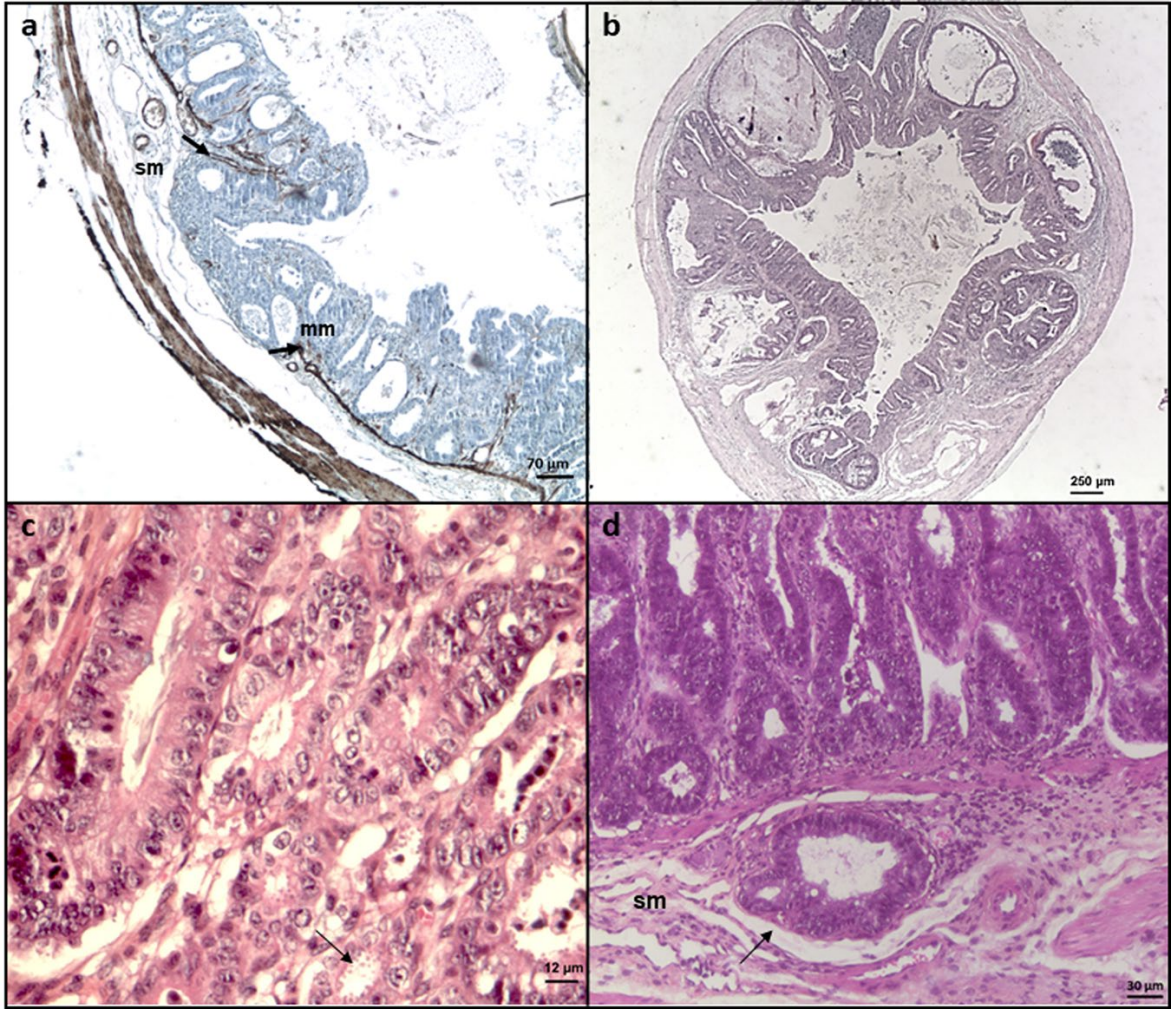
Histological sections of ileocecal regions of Dexamethasone-treated SCID mice infected with different *C. parvum* isolates. (**a**) *C. parvum* IOWA after 107 days post-infection (PI): presence of an invasive adenocarcinoma reaching the submucosa (sm) with an interruption (arrows) of the muscularis mucosae (mm) (immunohistochemical stain for alpha smooth muscle actin). Bar, 70 μm. (**b**) *C. parvum* DID after 62 days PI: presence of an adenocarcinoma invading the submucosa (hematoxylin and eosin staining). Bar, 250 μm. (**c**) *C. parvum* TUM1, after 19 days PI: high grade intraepithelial neoplasia characterized by epithelial atypia and associated with the presence of numerous parasites inside the glands (arrow) (hematoxylin and eosin staining). Bar, 12 μm. D) *C. parvum* CHR after 15 days PI: development of an adenocarcinoma (arrow) in the submucosa (sm) (hematoxylin and eosin staining). Bar, 30 μm.

The four IOWA, DID, TUM1 and CHR isolates of *C. parvum*, all identified as subtype IIaA15G2R1 after molecular identification, induced severe infections and gastrointestinal adenocarcinoma development in inoculated SCID mice (Fig. 1). To note, the time between *Cryptosporidium* isolation and mice inoculation and the oocyst viability were similar for all the experimental infections. Infectious dose was lower for the CHR isolate due to technical reasons. However, DID, TUM1 and CHR isolates were more virulent in this animal model. Particularly, the post-infection mortality rate at 60 days for mice inoculated with TUM1, DID and CHR isolates was 29, 41 and 29% respectively, while mice inoculated with *C. parvum* IOWA were all alive at this time (Table 1). Moreover, the time to develop a digestive neoplastic lesion was much faster for the 3 more virulent isolates (*e.g*. 45 days for the IOWA isolate *vs*. 15 days for the CHR isolate) and only these mice developed extra gastro-intestinal lesions (Table 1).

### Ion Torrent and Illumina sequencing

The sequencing run of DID and TUM1 isolates indexed on an Ion 318v2™ Chip resulted in approximately 1.66 GB of data with a mode reads of 369 bases. A total of 3,016,632 output sequence reads for DID and 2,921,126 for TUM1 with an average length of 272 and 284 bases per read respectively were obtained. After trimming, a total of 10,440,766 reads were obtained for the HiSeq Illumina sequencing (2 ×150 bp) of CHR isolate, of which 7,146,886 were concordant reads (*i.e*. properly aligned reads).

### **MICRA bio-informatic analysis of DID, TUM1 and CHR*C. parvum*isolates**

MICRA was first used in completely automatic way with bacterial reference sequences to filter out the contaminant bacterial reads of WGS data of DID, TUM1 and CHR isolates. Evidence of contamination from several bacterial species was only present in data from TUM1 isolate. For example, *Lactobacillus reuteri* (a lactobacillus naturally present in the gastrointestinal tract of mammals) genome was covered at more than 78% by the TUM1 sequence reads (see Suppl. File 1 in Suppl. File). Therefore, fastq files for DID and CHR isolates were not filtered and contained, respectively 3,016,632 and 10,513,932 reads whereas the fastq file for TUM1 isolate (2,921,126 reads) was filtered from bacterial reads leading to a file containing 2,654,324 reads. SNAP^22^(version 0.15) was used to identify the closest reference genome and the *C. parvum* IOWA genome came out as a result for DID, TUM1 and CHR isolates. All DID and TUM1 reads were thus mapped against *C. parvum* IOWA genome with SHRiMP2, an accurate mapper^23^(version 2.2.0). CHR reads were mapped against *C. parvum* IOWA genome with Bowtie 2^24^.

As seen in Table 2, SHRiMP2 or Bowtie 2 analyses revealed the presence of 849, 468 and 1076 SNVs between DID, TUM1 and CHR, respectively and the reference *C. parvum* IOWA genome. 55.9%, 49.1% and 57.2% of them occurred in coding regions from which 58.5%, 65.2% and 62.3% were non-synonymous SNVs (nsSNVs) (Table 2). DID, TUM1 and CHR reads which were not mapped against the *C. parvum* genome in this step were then mapped iteratively against the *C. hominis* and finally the *C. muris* genomes. Only two *C. hominis* CDSs were commonly found in DID, TUM1 and CHR sequences that were not found in *C. parvum* IOWA reference genome. No *C. muris* CDS was found in the remaining DID, TUM1 and CHR reads. The two *C. hominis* CDSs were Chro.60630 and Chro.60599, both belonging to subtelomeric regions of chromosome 6. After a blastn analysis of these two *C. hominis* CDSs, we found that they corresponded to sequences found in *C. parvum* chromosome 6, complete sequence; segment 4/4 (GI BX538353). A poor gene annotation of the *C. parvum* IOWA reference genome at this location could explain this result.

**Table 2:** SHRiMP2 and Bowtie 2 mapping statistics obtained from DID, TUM1 and CHR reads against the *C. parvum* IOWA genome.

*C. parvum* isolates	Coverage (%)	Mean sequencing depth	% mapped reads	#SNV	#SNV_CDS	#SNV_ change	#INDEL	#INDEL_CDS
DID	99.14	80.52	87.88	849	475	278	176	60
TUM1	98.44	53.76	65.18	468	230	150	165	58
CHR	98.76	139.72	82.63	1076	616	384	306	78

The first column represents the percentage of the *C. parvum* genome covered by at least 5 reads, the second column gives the mean sequencing depth and the third column shows the percentage of reads that mapped against the reference genome. The five last columns present the number of observed variants: #SNV is the total number of SNVs, #SNV_CDS is the number of SNVs in coding regions and #SNV_change is the number of variants which are non-synonymous. The total number of insertions/deletions is given by #INDEL and the number of indels located in coded regions is given by #INDEL_CDS.

At the end of the iterative mapping step, 918,741 reads were still unmapped for the TUM1 isolate, 340,965 reads for the DID isolate and 1,821,200 reads for the CHR isolate. The *de novo* assembly of the residual unmapped DID, TUM1 and CHR reads resulted in 10,476 contigs (of which 152 > 5,000 bp), 23,498 contigs (of which 226 > 5,000 bp) and 6,987 contigs (of which 3,339 > 500 bp) for DID, TUM1 and CHR, respectively. *De novo* contigs> 5,000 bp were then blasted against the nr database and results were parsed to only retrieve the organisms and size of the alignment. A semantic search for the terms “virus”, “onco”, “cancer”, “virulence” has returned no results. Various bacterial sequences have been found again in TUM1 data.

**Figure 2:**
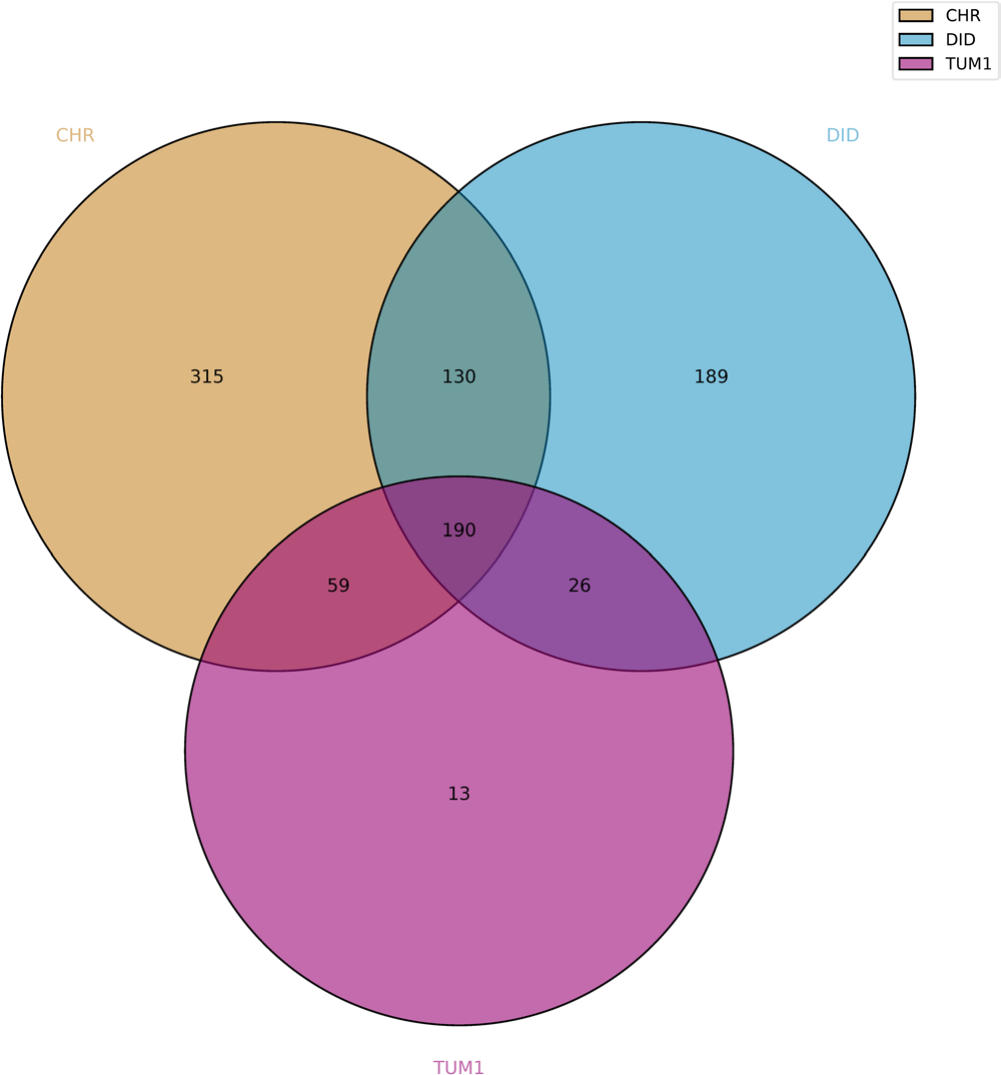
Venn diagram of common CDSs variants between DID, TUM1 and CHR isolates compared to *C. parvum* IOWA reference genome using the MICRA pipeline.

Finally, the MICRA comparison module identified a total of 190 variants in coding regions, corresponding to 126 CDSs, in common between the DID, TUM1 and CHR isolates compared to *C. parvum* IOWA reference genome (Fig. 2). These 190 variants correspond to 161 SNVs, 2 insertions and 27 deletions.

### **Custom bio-informatic analysis of DID, TUM1 and CHR*C. parvum*isolates**

**Figure 3:**
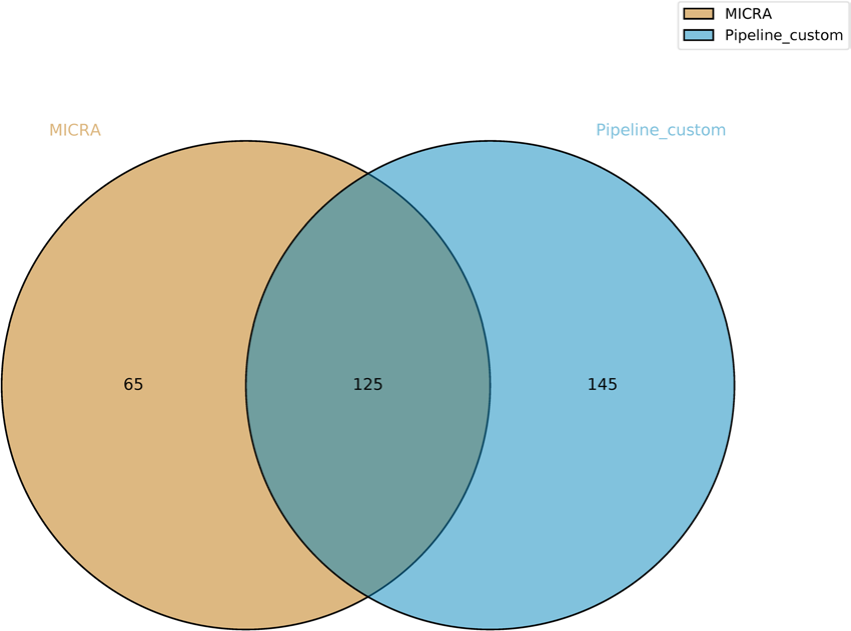
Venn diagram displaying the number of shared CDSs variants (*i.e*. common SNVs in CDSs between DID, TUM1 and CHR compared to IOWA) identified with MICRA and custom pipelines.

The bio-informatic analysis of DID, TUM1 and CHR reads was performed using a custom pipeline (see Methods). A bacterial contamination (mainly by *Lactobacillus* species) was also detected for TUM1 thanks to Kaiju program (Suppl. File 2 in Suppl. File). After mapping of the non-contaminated reads to *C. parvum* IOWA reference genome, a total of 1,136,427 mapped reads for DID, 766,759 mapped reads for TUM1 and 3,573,343 mapped reads for CHR were obtained, with a genome coverage breadth of 91.5%, 72.4% and 99% for DID, TUM1 and CHR isolates, respectively. Variant calling found 270 common variants on CDSs between the three isolates, compared to MICRA that found 190 common variants on CDSs between these strains (Suppl. Table S1). In total, 125 common SNVs were identified between TUM1, DID and CHR by the two methods (Fig. 3). To note, none of the common indels found by MICRA in DID, TUM1 and CHR were found by our custom pipeline (Suppl. Table S1). Thus, for the remaining of the study, we focused on these 125 SNVs that represent common variants on CDSs detected by the two bio-informatic pipelines between DID, TUM1 and CHR in comparison with *C. parvum* IOWA, the reference genome.

### **Analysis of the 125 common SNVs between DID, TUM1 and CHR*C. parvum*isolates**

A total of 125 SNVs of interest were associated with 90 *C. parvum* coding sequences (Table 3). Of these 125, 83 SNVs (found in 68 CDSs) were classified as non-synonymous. Only 6 variations on the 5 following genes cgd2_1400 (hypothetical protein); cgd2_450 (also known as CpMuc7, a mucin-like glycoprotein part of the seven mucin genes clustered on a single locus on chromosome 2^25^); cgd3_1690 (hypothetical protein); cgd5_860 (hypothetical protein) and cgd5_3210 (a large hypothetical protein with signal peptide) were predicted as deleterious by PROVEAN. Moreover, 2 other variants on CDSs cgd5_2290 (a hypothetical protein with signal peptide) and cgd6_5520 (an insulinase-like peptidase with a signal peptide) were predicted to have a high impact on protein function, *i.e*. a stop lost (Table 3). Finally, of the 125 common SNVs, 81 appear to have a moderate impact on protein function and are all missense variants (Suppl. Table S1).

**Table 3:** Characteristics of the 125 common SNVs found between *C. parvum* DID, TUM1 and CHR isolates in comparison with *C. parvum* IOWA.

	SNVs	Mutated genes	Non-synonymous SNV	synonymous SNV	SNV eliminating start codon	SNV causing premature termination codon	SNV eliminating termination codon	Non-synonymous SNV identified as deleterious by PROVEAN
Chr. 1	10	8	6	4	0	0	0	0
Chr. 2	12	10	9	3	0	0	0	3
Chr. 3	11	9	8	3	0	0	0	1
Chr. 4	11	8	9	2	0	0	0	0
Chr. 5	21	16	16	5	0	0	1	2
Chr. 6	9	8	6	3	0	0	1	0
Chr. 7	39	21	21	18	0	0	0	0
Chr. 8	12	10	8	4	0	0	0	0
Total	125	90	83	42	0	0	2	6

To test our results, verification by Sanger sequencing was performed on regions of 8 randomly selected CDS encompassing SNVs of interest for DID and TUM1. For all regions, obtained sequences were consistent with the MICRA and custom pipeline analyses of Ion Torrent sequencing results, validating therefore the 16 identified SNVs (Suppl. Table S2). Also, all of the 125 SNVs were already identified, at least once, in CryptoDB database (http://cryptodb.org/) for other *C. parvum* isolates like the anthroponotic *C. parvum* isolate TU114^7^ and/or *C. parvum* UKP isolates 2 to 8^9^.

Interestingly, when trying to identify hotspots of variation, we found that a lot of SNVs detected in this study were in the subtelomeric regions of chromosomes 1, 3, 5 and 6 as shown in Suppl. File 3 (in Suppl. File).

Blast2GO version 4.1.9 was used to assign GO terms to the annotated proteins of interest. To note, on 3,805 total genes for *C. parvum* IOWA, only 2,223 were Blast2GO annotated, and on 90 genes of interest, only 58 were Blast2GO annotated. Briefly, a BLASTX-fast search to the nr database was carried out, the accession numbers were mapped to the Gene Ontology database and only those with an e-value lower than 1.10^−6^ were kept.

**Figure 4:**
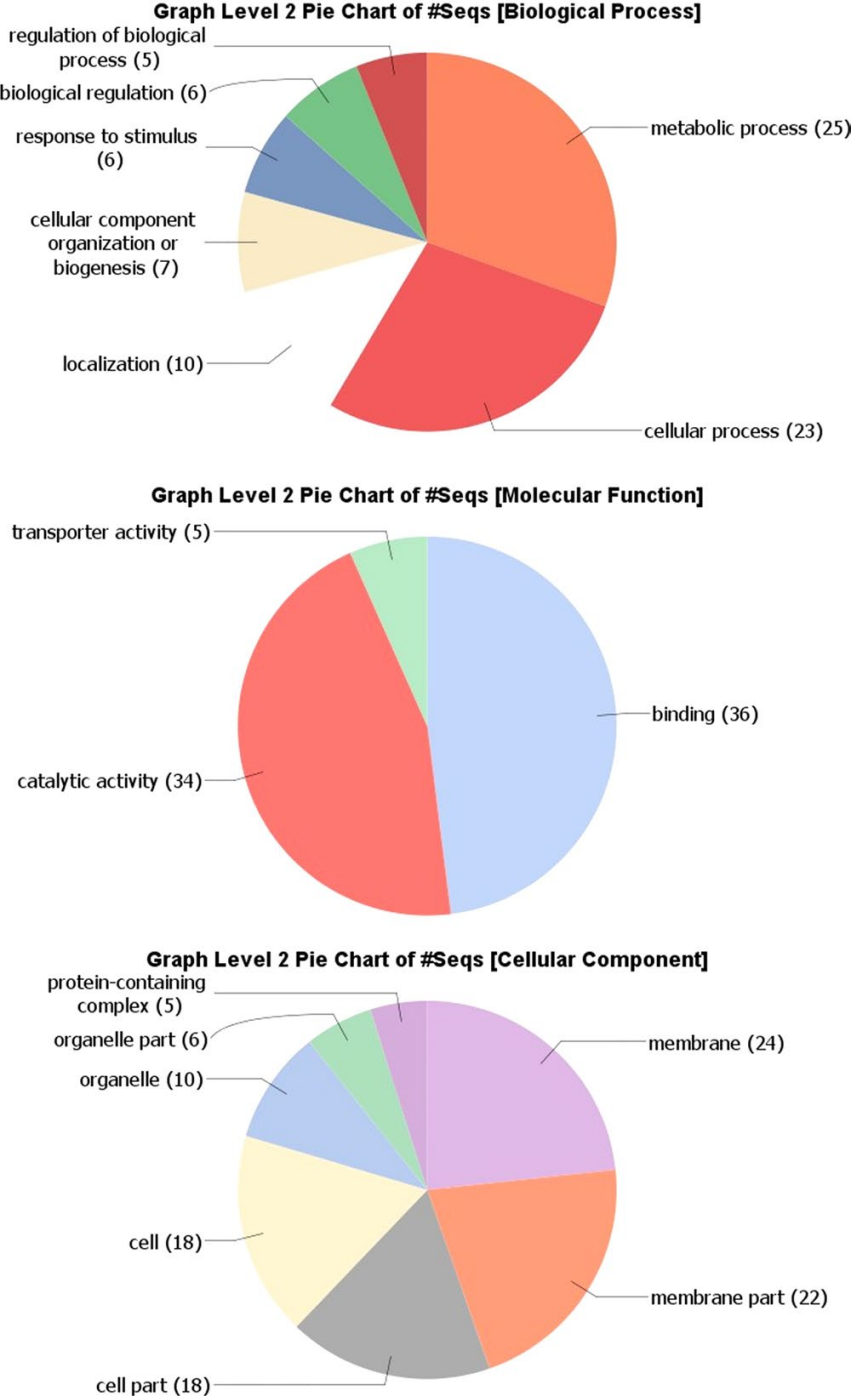
Blast2GO analysis of 90 CDS of interest (encompassing common SNVs found between DID, TUM1 and CHR isolates). Combined graphs were performed in Blast2GO at level 2 for Molecular Function, Cellular Component and Biological Process aspects of Gene Ontology. Values within parentheses are the number of sequences associated with each GO term.

The Blast2GO analysis showed that CDSs of interest are mostly involved in metabolic and cellular processes, while the molecular functions are clearly divided between binding (48%) and catalytic activity (45%) (Fig. 4). The Blast2GO analysis of cellular components revealed that a large number of SNVs are identified in membrane proteins (Fig. 4). Further investigation of the metabolic and cellular processes revealed organic substances, primary, cellular and nitrogen-compound metabolic processes at level 3, each accounted for 14–17% of the total number of sequences (Suppl. File 4 in Suppl. File). Concerning molecular functions, GO level 3 showed ion, protein, organic cyclic compound, heterocyclic compound, small molecule, carbohydrate derivative and drug binding as well as hydrolase activity, catalytic activity (acting on a protein) and transferase activity (Suppl. File 4 in Suppl. File). Finally, the level 4 pie chart for the cellular component indicated that 51% of the sequences were classified as integral component of membranes GO, followed by intracellular membrane- or non-membrane-bounded organelles and cytoplasm GO (Suppl. File 4 in Suppl. File).

Interestingly, Kyoto Encyclopedia of Genes and Genomes (KEGG) pathway analysis in Blast2GO suggested that some of these genes of interest are involved in Phosphatidylinositol signaling system, purine, arginine and proline metabolism, lysine degradation (histone-lysine N-methyltransferase encoded by cgd5_400), amino sugar and nucleotide sugar metabolism, Th1 and Th2 cell differentiation and T cell receptor signaling pathway (serine/threonine-protein phosphatases encoded by cgd3_250, cgd7_4470 and cgd6_3570) (Suppl. Table S3). Particularly, a substantial number of phosphatases was recovered like cgd2_230 (a Phosphatidylinositol_phosphate_phosphatase); some nucleoside triphosphatases, cgd6_3570 (a Protein-tyrosine-like/Myotubularin-like_phosphatase_domain_containing_protein), cgd3_250 (a protein serine/threonine phosphatase alpha, from PP2A family) and cgd7_4470 (a CDC14 phosphatase) (Suppl. Table S3).

All results of Pfam, SMART, balstp, blastx searching against nr database in GenBank analyses are compiled in Suppl. Table S1. Moreover, SignalP predicted 16 CDSs of interest with signal peptides (Suppl. Table S4). Trans-membrane (TM) domains were also found in cgd5_270 and cgd7_1560. SMART analysis also predicted that cgd5_280, cgd6_1180 and cgd7_4530 had a signal peptide. SignalP only has found TM domains in these CDSs. In GPISom, only one CDS, *i.e*. cgd3_3520, appears as a GPI-anchored protein. Another one, cgd8_4190 appears as “undecidable sequence”. To summarize, we found a large number of variants in *C. parvum* proteins with a signal peptide.

Then, we inspected in the literature the genes already described as involved in *Cryptosporidium* virulence and searched our 90 CDSs of interest. Particularly, we looked for virulence genes described in Bouzid *et al*., 2013^15^ and the ProtVirDB database. Only four gene families were identified in our study out of all the genes already described and implicated in *Cryptosporidium* virulence. These families were as follows: mucins (cgd2_450 and other predicted mucins like cgd3_720, cgd5_340, cgd7_5440 and cgd8_660), ATP-binding cassette (ABC) transporters (cgd4_4440, cgd7_4510 and cgd7_4520), ATPase3 (cgd3_1110) and cysteine proteases (cgd2_3450; cgd8_1320 and cgd7_2760) encoding genes. All these proteins have been suggested previously to be implicated in attachment/invasion (mucins) or intra-cellular multiplication/survival (ABC transporters, ATPase3 and cysteine proteases) of *Cryptosporidium* developmental stages in the host^15^. However, it is not obvious to establish whether *Cryptosporidium* proteins are specific for a well-defined developmental stage^26^. Indeed, when looking for the expression profiles of the 90 proteins of interest during the life cycle of the parasite (Widmer and Lippuner RNAseq datasets in CryptoDB), we showed that most genes were mainly overexpressed in the intracellular stages of *Cryptosporidium*, but that some of them were overexpressed in the oocyst or sporozoite stages (like cgd2_340 coding for a signal peptide large protein, cgd7_4510 coding for an ABC transporter and cgd3_720 coding for a mucin protein) (Suppl. Table S5).

## Discussion

In this study the genomes of three highly virulent *C. parvum* isolates isolated from fecal samples of naturally infected animals or humans and reported to induce digestive adenocarcinoma in a rodent model^17–21^, were sequenced and compared with the reference genome *C. parvum* IOWA^5^. Briefly, the laboratory *C. parvum* IOWA isolate was shown to be able to induce a chronic infection and the development of invasive digestive adenocarcinoma even with very low inoculum in immunocompromised mice. Three other *C. parvum* isolates of animal (TUM1) or human (DID and CHR) origin were also able to induce a durable infection and the development of neoplasia in the same murine model. However, the isolates DID, TUM1 and CHR were more virulent than the IOWA isolate in terms of severity of infection, time of onset of neoplastic lesions and mortality (Table 1). As our main goal was to contribute to the understanding of the varying virulence of these carcinogenic *C. parvum* isolates, our genomic analysis was based on the study of potential genetic differences among isolates that could explain these differences. We focused on sequence polymorphisms because previous inter- or intra-species genomic comparative studies have shown an almost perfect synteny of the genomes of *C. parvum* and *C. hominis* strains and have suggested that phenotypic differences between these strains should be linked to subtle sequence differences such as SNVs or indels^14^. For example, Isaza *et al*.^10^ found 152 SNVs including coding and non-coding regions when they compared the genomes of *C. hominis* TU502 new and *C. hominis* UKH1, two isolates of subtype Ib family based on GP60 gene sequence. To note, in our work, the three sequenced isolates of *C. parvum* belong to the IIa subtype family, as the IOWA isolate.

Before sequencing, oocysts were isolated directly from field specimens and purified by IMS. Hadfield *et al*.^9^ have already shown the superiority of IMS over cesium chloride density centrifugation to properly purify *Cryptosporidium* oocysts before sequencing, and reduce contaminant DNA levels. In order to generate enough DNA material for sequencing, *C. parvum* genomes DNA were subjected to a WGA. This technique could favor amplification bias in some degree, resulting in non-random genome coverage and erroneous DNA sequences. However, we used here an approved MDA method with ɸ29 DNA polymerase to limit these biases^27^. Moreover, different verifications confirmed that this technique did not affect significantly the outcome of our comparative genomic analysis. First, the MICRA pipeline revealed that *C. parvum* IOWA genome was covered at >98% by reads of DID, TUM1 and CHR isolates. Furthermore, Sanger sequencing was performed on regions of randomly selected CDS encompassing SNVs of interest and confirmed the presence of these SNVs. Finally, all of these SNVs were already identified in CryptoDB database (http://cryptodb.org/) for other *C. parvum* isolates (like the anthroponotic *C. parvum* isolate TU114^7^ and/or *C. parvum* UKP isolates 2 to 8^9^).

Unlike analysis of data from bacteria, there are no established pipelines for comparative genomic analyses of WGS data from parasites. MICRA^28^, a pipeline initially developed to identify and characterize bacterial genomes through high throughput sequencing reads analysis, was successfully used here for the first time to analyze eukaryotic genomes. However, in order to give more weight to the results obtained with MICRA, we decided to test another custom pipeline using, among others, BFCTools. Also, aware that false positives are common in comparative genomics studies, the analytical approach implemented in this work aimed to radically limit these false positive mutations that can not only distort the picture of a genomic subject, but also generate significant additional costs and analytical time if their amount is substantial. This robust comparative genomics approach, focusing on specificity rather than sensitivity, allowed us to limit the number of these false positive mutations to be investigated. Indeed, 100% of the mutations tested in Sanger sequencing as a standard gold technique have been validated and the 125 SNVs have already been described at least once in the *C. parvum* genomes available in CryptoDB.

A total of 125 SNVs, validated by two independent pipelines and shared between the three highly virulent isolates in comparison with *C. parvum* IOWA genome, were found. These 125 SNVs were associated with 90 *C. parvum* coding sequences. In contrast to the results already reported by Feng *et al*.^8^ who compared three *C. parvum* isolates and found that 61.8–63.2% of the SNVs occurred in coding regions, we found 49.1–57.2% of SNVs located in coding regions. Also, Isaza *et al*.^10^ found 62–65% of non-synonymous substitutions between various *C. hominis* isolates but only 48% of nsSNVs when comparing *C. hominis* TU502 “new” and *C. hominis* UKH1, while we detected 66.4% of nsSNVs in our study. Besides these 83 nsSNVs described here, a total of 42 synonymous SNVs were found. It has long been assumed that synonymous SNVs are insignificant. However, a number of recent studies have challenged this hypothesis, showing that synonymous mutations are also under evolutionary pressure and may be involved in disease. In the human genome, some studies have revealed that synonymous polymorphisms can affect splicing, stability and structure of messenger RNA and protein folding and thus have a significant effect on protein function^29^. Therefore, it seemed important to us to study all the 125 SNVs, impacting 90 genes in our analysis. When studying the Gene Ontology of these 90 genes of interest, only 58 were Blast2GO annotated. Interestingly, a large number of these genes were involved in binding and catalytic activity, and half of them were coding for membrane proteins. Besides, more than 20 genes seemed to be destined towards the secretory pathway, as they exhibited a signal peptide (Suppl. Tables S1, S4). It is worth considering that of these 90 genes of interest, the majority are over-expressed in the intracellular stages of the parasite, although some are over-expressed in the oocyst and sporozoite stages (Suppl. Table 5). These results confirm that beyond the genes involved in intracellular maintenance and damage to the host cell, genes involved in the initial interaction processes of *Cryptosporidium* oocysts and sporozoites with host epithelial cells can also be considered as virulence factors of the parasite.

Four families of genes identified in this work were already described to be implicated in parasite virulence and parasite-host interaction^15^, namely mucins, transporters (ABC and ATPase3) and cysteine proteases.

Concerning mucins, these proteins are known for their implication in sporozoite attachment and invasion of the epithelial cell, and for their high immunogenicity naturally leading to their gene sequence polymorphism^25^. In addition, mucin type glycoproteins have been proposed as potential determinants for differences in host range among *Cryptosporidium* species and genotypes potentially playing a role in tissue tropism and virulence^13^. In this work, most of the mucin genes found interesting due to their polymorphism were mostly predicted mucins from various chromosomes. With the exception of CpMuc7 (cgd2_450), none of them belonged to the well-known seven mucin genes clustered in chromosome 2^25^.

Besides, the three *C. parvum* isolates sequenced in this study differed from *C. parvum* IOWA in three genes coding for ABC transporters. Thirty three *Cryptosporidium* ABC transporters have been estimated^30^, 13 of which have been identified according to TransportDB database (http://www.membranetransport.org). These ABC transporters have been described by others as highly divergent genes^7^. Members of this family are mainly recognized to be implicated in multidrug resistance (MDR) and resistance-associated protein (MRP). However, ABC transporters can also be involved in cellular processes like DNA repair, translation or regulation of gene expression^31^. For bacteria, ABC transporters are associated with pathogenesis or virulence^32^ and some of them could participate to the process of adhesion or invasion of cells^33^. In humans, it has also been shown that the exposure of fibroblasts to ATP binding cassette transporter A1 (ABCA1) ligands like Apolipoprotein A-I results in the generation of intracellular signals, including activation of the small G-protein Cdc42, protein kinases (PAK-1 and p54 JNK), and actin polymerization^34^. Consistently, different investigations have reported that *C. parvum* induces actin reorganization at the sites of infection by modulation of different signaling pathways including for instance, the activation of the small GTPase Cdc 42^35^ but the potential implication of ABC transporters in this process is not known.

Recently, published works have shown that some *C. parvum* transcripts can be selectively delivered into epithelial cells during infection and may modulate gene transcription in infected host cells^36–39^. For example, Wang *et al*.^36^ have described that the delivery of parasite Cdg7_Flc_0990 RNA transcript into intestinal epithelial cells during *C. parvum* infection suppresses host cell gene transcription through epigenetic mechanisms. Interestingly, when blasting this Cdg7_Flc_0990 sequence, we found 100% of similarity with cgd7_4800 mRNA. This cgd7_4800 gene is not in our list of genes of interest but it codes for an ABC transporter protein. Therefore, it will be very interesting to study whether the transcripts of the ABC transporters proteins found in our study could play a similar role. According to Sauvage *et al*.^31^, cgd7_4510 and cgd7_4520, two out of our three ABC transporters encoding genes of interest, may be involved in the antifolate resistance, but the cgd4_4440 gene has an unknown function to date. More generally, the study of parasitic molecular effectors that can be transmitted to infected host cells and play a role in the pathogenesis of diseases seems to be of major interest to decipher the physiopathology of infections induced by these carcinogenic *C. parvum* isolates.

Of the three cysteine proteases of interest found in this work, one of them, *i.e*. cgd8_1320, a calpain-like protein, is particularly interesting because members of the calpain family are believed to function in various biological processes including integrin-mediated cell migration, cytoskeletal remodeling, cell differentiation and apoptosis^40,41^. Since its precise function is currently unknown in *Cryptosporidium*, its study deserves further research.

Besides these families of *Cryptosporidium* virulence factors already described in the literature^15^, our study identified new potential virulence factors in carcinogenic *C. parvum* isolates. Among them various phosphatases in which a large number of variants were found. Particularly, the cgd3_250 gene has caught our attention. Indeed, the SMART analysis of cgd3_250 revealed that it contained 2 Kelch domains as well as the catalytic domain of a protein phosphatase 2 A (PP2A), which belongs to the large serine/threonine phosphatase family. Interestingly, PP2A activity takes part in the majority of the cellular pathways in many eukaryotic systems and its dysfunction or deregulation will affect various physiological processes such as cell proliferation, signal transduction and apoptosis. PP2A also plays a major role in the Wnt signaling pathway and is considered as a tumor suppressor^42^. To note, the role of another serine-threonine phosphatase type 2 C (TgPP2C) in the *Toxoplasma gondii* - host cell interaction has already been described^43^. This TgPP2C is involved in regulating host cell apoptosis through an inhibitory effect^43^.

The Cgd4_4470 gene coding for a Cdc14 phosphatase has also attracted our attention. These Cdc14 phosphatases have been studied previously only in yeasts and metazoans. Ccd14 is an essential dual-specificity phosphatase that counteracts Cdk1 activity during anaphase to promote mitotic exit in *Saccharomyces cerevisiae*. However, in humans, CDC14A is not essential for cell cycle progression but it regulates cell migration and cell adhesion^44^. Particularly, Chen *et al*.^45^ found a reduction in catenin enrichment (α/β catenin) at cell-cell junctions and a decrease in E-cadherin levels when hCDC14A phosphatase activity was eliminated or when the eplin (a tumor suppressor and substrate of hCDC14A) was removed. In addition, a reduction in the levels of *hCDC14A* and *eplin* mRNA is a common feature of colorectal carcinoma and is associated with poor prognosis. Thus, the authors concluded that this loss of regulation of hCDC14A-eplin may be a key step in the evolution of invasive colorectal cancer and that hCDC14A may directly contribute to the metastatic potential of tumors^45^.

In this study, we also found one SNV in cgd5_400, annotated in CryptoDB as a Histone-lysine N-methyltransferase. Protein lysine methyltransferases (PKMTs) are a group of proteins involved in post translational modification (PTMs) that can catalyze the transfer of methyl groups from the cofactor S-5′-adenosyl-L-methionine to lysine residues of histone and non-histone substrate. The PTMs of histone are epigenetic regulations that dynamically control diverse biological process including the regulation of gene expression and transcription, which affect cell proliferation and differentiation, cell migration and invasion. For example, some epigenetic modifications like histone post-translational modifications in host–pathogen interactions were described to be implicated in virulence of some parasites like *P. falciparum* or *E. histolytica*^46^. Numerous studies have also associated these enzymes as critical determinants for tumor initiation and progression. The Val(795)->Ile mutation found in our study in cgd5_400 is not in the SET domain of the PKMT, a conserved domain essential for the catalytic activity of histone lysine methyltransferases.

In their study in 2015, Isaza *et al*.^10^ have found five protein-coding genes from *C. parvum* IOWA that were absent in the *C. hominis* UdeA01 genome sequenced by these authors. From these 5 genes, two were present in the 90 CDSs of interest in our study: cgd6_5510 and cgd6_5520, two telomeric insulin-like peptidases (LuxS/M16 peptidase-like metalloenzymes). Guo *et al*.^11^ have suggested that duplication and interallelic recombination of telomeric genes like the two cgd6_5510 and cgd6_5520 could be the cause of the host expansion in *C. parvum*. Interestingly, two other members of M16 family metalloproteases called toxolysin 1 and 4 have been shown to be rhoptry or microneme-associated in *Toxoplasma gondii* and could be involved in cell invasion^47,48^. Other difference in copy numbers of MEDLE or SKSR families secreted proteins were reported to be involved in *C. parvum* host specificity^8,11^. In their comparative genomic analysis between IIa and IId *C. parvum* isolates, Feng *et al*.^8^ found that most of the SNVs detected were in subtelomeric regions of chromosomes 1, 4 and 6. In our study, hotspots of variation were also identified in subtelomeric regions of chromosomes 1, 3, 5 and 6. These subtelomeric genes include those encoding SKSR secretory proteins, the MEDLE family of secretory proteins, and insulinase-like proteases. Here, no MEDLE coding genes were found in our 90 CDSs of interest but a SNV was encountered in cgd3_10 (SKSR).

In conclusion, we present here the first comparative genomic analysis of four carcinogenic *C. parvum* isolates with varying virulence. Besides already described virulence factors in *C. parvum* genome, new potential virulence factors were identified in this study. Many of these genes code for membrane proteins, appear to be destined towards the secretory pathway or have been implicated in the cytoskeleton remodeling. Interestingly, it is well known that some virus, bacteria and parasites, are able to influence signaling pathways that regulate the cytoskeleton function, being the rearrangements of the actin cytoskeleton crucial to optimize their biological cycles^49^. Consistently, it was reported previously in the mouse model of digestive carcinogenesis induced by *C. parvum*, that the Wnt pathway, and the cytoskeleton network were modulated and seemed to be pivotal for the development of the neoplastic process^21^. However, it is generally believed that the infected intestinal cells harboring *Cryptosporidium* are destined to die after the egress of the parasite. How these oncogenic isolates may thus transform normal cells into transformed cells? One explanation could be that even if *Cryptosporidium* induces signaling events locally at the site of infection, the activation of these signaling pathways will probably have global consequence for the whole cell, and eventually for the entire tissue and cytoskeletal architecture^35^. In addition, it has been described that oncogenic pathogens are able to hijack the cell cycle checkpoints inducing genomic instability, increasing the life span (i.e. inhibiting apoptosis) and subverting senescence. Cells that accumulated genetic and epigenetic lesions are stimulated to proliferate, and the accumulation of lesions in a given lineage perhaps gives rise to a cell clone^50^. Particularly, *Theileria*, another apicomplexan protozoan as *Cryptosporidium*, is able to induce uncontrolled proliferation and transformation of host cells^51^. Taken together, the CDSs found in the newly sequenced genomes of *C. parvum* isolates when compared with that of reference genome could explain the difference in virulence. However, the mechanisms by which f *C. parvum* is able to induce transformation of the host cells are still unknown. The new targeted genome editing tools like CRISPR/Cas9 can enable us to study the biological function of these genes of interest in the parasite and to test their implication in the virulence and/or carcinogenic potential. Whatever the potential medical impact of this carcinogenic process in humans, the study of the *Cryptosporidium* virulence factors provides clues to understand host-parasite interactions. Further studies are needed to understand the pathogenicity of this parasite which is highly tumorigenic when inoculated in an animal model, and to substantiate additional links with cancer induction.

## Materials & Methods

### *C. parvum* isolates

The *C. parvum* TUM1 isolate was isolated from a calf in USA and was kindly provided by Donna Akiyoshi and Saul Tzipori, from Tufts Cummings School of Veterinary Medicine (Boston, USA)^18^. The *C. parvum* DID isolate was recovered from stool samples of a 51-year-old man with acute lymphoblastic leukemia who nearly drowned in the Deûle River (Lille, France) some weeks after undergoing an allogeneic stem cell transplantation. He developed a fulminant cryptosporidiosis only two days after being rescue^20^. The *C. parvum* CHR isolate was recovered in France from stool samples of an immunocompetent 19-year-old woman with diarrhea, previously involved in milking dairy cows for 2 months (unpublished data). Authorization for utilization of the stool isolates that were collected in Lille University Hospital was obtained from the French Ministry of Research (N°DC-2008–642). The requirement for informed consents was waived because the experiments did not result in additional constraints for the patients. Moreover, all the methods used in the study were carried out in accordance with the approved guidelines (World Medical Association’s (WMA) Declaration of Helsinki-Ethical Principles for Medical Research Involving Human Subjects). The *C. parvum* IOWA isolate was commercially available at Waterborne™, Inc. (New Orleans, Louisiana) after several passages through calves.

For molecular identification of the *Cryptosporidium* isolates a fragment of the 18 S rRNA gene was amplified by nested PCR^52^ and sequenced. A subtyping based on sequence analysis of the GP60 gene was performed^53^. In order to rule out the presence of other pathogens in the inoculum, the absence of bacteria or fungi was assured by testing the oocyst suspensions on Plate Count Agar and on Sabouraud plates at 37 °C for 1 week.

### **Phenotypic characterization of*Cryptosporidium parvum*isolates from inoculated immunosuppressed mice**

The phenotypic characterization of infection by DID and TUM1 isolates of *C. parvum* in our SCID mouse model has already been described elsewhere^18–20^. The same experimental mouse model, inoculation conditions, histopathological and immunohistochemical protocols were used to characterize the more recently sequenced *C. parvum* CHR isolate (unpublished data).

In order to evaluate the virulence of each *C. parvum* (IOWA, DID, TUM1 or CHR) isolate inoculated to SCID mice, we considered the clinical signs in mice and their mortality rates (Table 1). Also, we focused on the kinetic anatomopathological study of organs in which neoplastic lesions usually develop during *C. parvum* infections (*i.e*. gastric antrum, caecum, bile ducts of the hepatic hilum)^18–20^ and that were collected after euthanasia at specific times post-infection (Table 1). Briefly, organs were removed fixed in 10% buffered formalin and processed using standard staining technique like HES. The Volgens-Gomori stain was employed for assessing the gland membrane integrity. An anti-cytokeratin monoclonal antibody (AM071-5M, Biogenex, Netherlands) was used to evaluate the invasion of epithelial cells into the *lamina propria* and in deeper organ layers. Anti-alpha smooth muscle actin monoclonal antibody (M0851, Dako, Denmark) was used to visualize the *muscularis mucosae* disruption or the *muscularis* penetration by neoplastic glands. To clarify the histological severity of neoplastic lesions ranging from low-grade dysplasia to invasive adenocarcinoma, we referred to the human nomenclature and the Vienna classification of intra-epithelial neoplasia (dysplasia) and related lesions^54^, the World Health Organisation (WHO) classification of tumors^55^ as well as the Consensus Report and Recommendations for Pathology of Mouse Models of Intestinal cancer^56^. SCID mice were obtained from the animal facility of the Institut Pasteur de Lille (research accreditation number, A59107) from a colony regularly controlled and known to be free of pathogens including *Helicobacter*. Animals were maintained under aseptic conditions in an isolator with standard laboratory food and water *ad libitum*. Animal protocols were approved by the French regional ethical committee (approval number CEEA 112011). All methods were performed in accordance with the relevant guidelines and regulations.

### Parasite purification

Oocysts were purified respectively from feces of *C. parvum* TUM1 infected mice^18^ and *C. parvum* DID and CHR infected patients using IMS technique using the anti-*Cryptosporidium* Dynabeads®kit (Life Technologies). At least ten IMS experiments were performed for each isolate. One hundred microliters of Dynabeads® anti-*Cryptosporidium* were incubated with 400 µL of patient feces suspension or infected mice hydrated feces at room temperature (25 °C) in Buffers A and B using a rotating mixer (Dynabeads® Sample Mixers, Life Technologies) for 60 min. After immunocapture of oocysts, the bead–parasite complexes were magnetically separated from the suspension and debris using a magnetic particle concentrator (Dynabeads® MPC®-1, Invitrogen) for 2 min. After that, the bead-parasite complexes were washed twice with 1 mL of Buffer A 1X and then 100 μL of 0.1 N HCl were added to disrupt these complexes. Using a magnetic particle concentrator (Dynabeads® MPC®-S, Invitrogen) parasites were magnetically separated from beads and finally 10 µL of NaOH 1 N were added to the purified parasite solution. For each isolate, ten IMS products were pooled in order to perform the DNA extraction.

### DNA extraction and Whole Genome Amplification (WGA)

DNA was isolated from 200 μL of each purified parasite suspension using the QIAamp DNA minikit (Qiagen, Hilden, Germany) following the manufacturer’s instructions. The extracted DNA was eluted with 45 μL of elution buffer and stored at −20 °C before use. MDA was performed with Illustra GenomiPhi V2 DNA Kit (GE HealthCare). Since no lysis was applied, samples were directly amplified for 2 h at 37 °C and Phi 29 enzyme was inactivated by heating 10 min at 65 °C. Amplified DNA (aDNA) were purified by QIAamp DNA mini kit (QIAGEN, Hilden, Germany) according to supplier recommendations, eluted in 50 µL, TE 1×, pH 8 then quantified with Quant-IT picogreen dsDNA Assay kit (Life Technologies, Carlsbad, USA). Samples were fragmented to 400 bp size by Ion Plus Fragment Library Kit (Ion Torrent, Life technologies) according to supplier recommendations. Fragmentation quality was evaluated by High sensitivity DNA Kit (HS Kit) on 2100 Bioanalyzer (Agilent technologies).

### Ion Torrent and HiSeq Illumina sequencing

The WGA products from DID and TUM1 isolates were used to generate libraries and sequenced on a PGM™, Ion Torrent (Life Technologies). Briefly, fragmented DNA were ligated with sequencing barcoded adapters using Ion-Xpress barcode adapters 1–16 kit (Ion Torrent, Life Technologies). A DNA size selection was performed using E-gel size select 2% (Invitrogen, Carlsbad, USA) to retrieve fragments around 450 bp and each library was monitored using HS kit. Both libraries were equimolarly pooled then adjusted to 25 pM. Indexed libraries were clonally amplified with Ion PGM™ Template OT2 400 Kit and the Ion OneTouch™ ES Instrument (Ion Torrent, Life Technologies) according to supplier recommendations to obtain an enrichment in template-positive Ion PGM™ Template Ion Sphere™ Particles (ISP). Then, 30 µL of ISP suspension (*i.e*. template for DNA sequencing) were introduced in Ion 318™ Chip Kit v2 (Ion Torrent, Life Technologies) to proceed to high throughput sequencing using PGM Ion Torrent Benchtop sequencer and Ion PGM™ Sequencing 400 Kit (Ion Torrent, Life Technologies). All PGM quality-approved, trimmed, and filtered data obtained by using CLC Assembly Cell 4.1.0 were exported as SFF files.

The WGA products from CHR isolate were fragmented, ligated to Illumina adapters and the library was sequenced on a HiSeq. 2500 platform (Illumina) (2 × 150-bp).

### Bio-informatic analysis

In order to identify common CDS_SNVs between the three most virulent *C. parvum* strains, the main objective of the analytical approach used in this work was to limit the amount of false positive mutations regularly observed in comparative genomic analyses. Thus, the output data from the two sequencers (Ion Torrent read sequences for DID and TUM1 and HiSeq read sequences for CHR) were analyzed in parallel by two different bioinformatic approaches: an integrated and automated one (MICRA), and an other carried out *ad hoc*.

### MICRA analysis

A recently published automatic WGS data analyzing pipeline, MICRA^28^, was firstly used with the Ion Torrent and HiSeq sequencing data to characterize and compare the *C. parvum* DID, TUM1 and CHR genomes. In a first step, MICRA was used in completely automatic way with bacterial reference sequences to filter out the contaminant bacterial reads. The residual reads were then used to build the genomic comparative analysis. As MICRA was developed for bacterial data, meaning that a unique chromosome is only considered as reference sequence, the chromosomes of the *Cryptosporidium* reference genomes (*i.e. C. parvum* IOWA AAEE00000000.1, *C. hominis* TU502 NZ_AAEL00000000.1 and *C. muris* RN66 AAZY00000000.2) were concatenated and the corresponding concatenated FASTA and GFF files were used as input of MICRA. In the first step of MICRA, a very fast mapper, SNAP^22^(version 0.15), was used to identify the closest reference genome for the three strains, which resulted to be *C. parvum* IOWA. Then, the sensitive SHRiMP2 program^23^(version 2.2.0) or Bowtie 2^24^ was used to map the complete set of reads against *C. parvum* IOWA genome and calculate various mapping statistics. At this step several files were generated: one file containing all SNVs and indels found between DID, TUM1 or CHR and *C. parvum* IOWA (generated after variant calling using the following parameters: minimum 5 mapped reads at the position of interest and minimum variant frequency of 0.9); files containing the consensus sequences and consensus CDS sequences generated from mapping against *C. parvum* IOWA for DID, TUM1 and CHR isolates. In a third step, an iterative mapping of DID, TUM1 and CHR reads against *C. parvum*, then *C. hominis* and finally *C. muris* genomes was performed in order to identify DID, TUM1 and CHR specific sequences that were not found in *C. parvum* IOWA reference genome but potentially present in *C. hominis* or *C. muris* genomes. The final step of the pipeline consisted in a *de novo* assembly of the remaining unmapped reads after the iterative mapping step using MIRA^57^(version 3.9.16). *De novo* contigs were then blasted against nr database to identify previously undetected genes. Complementary modules of the MICRA pipeline were finally used to quickly compare the lists of covered CDSs and the lists of variants between TUM1, DID and CHR isolates, allowing us to identify the common CDSs and variations between the three isolates.

### Custom bioinformatics pipeline

In parallel, a custom bioinformatics pipeline has been developed, that was used in a complementary way to MICRA to reinforce the results obtained for the Ion Torrent DID and TUM1 reads and Illumina CHR reads. First, raw sequencing reads were cleaned and quality controlled with Trimmomatic 0.36^58^ and FastQC^59^, respectively. To note, FastQC was used with ‘Q = 20′ on Ion Torrent reads and with default parameters on Illumina reads. Then, Kaiju^60^ was used to detect microbial contaminants in whole genome sequencing data. Corresponding reads were removed after being mapped on the bacterial genomes found by Kaiju with Bowtie 2 v2.2.6^24^.

For all three datasets, the non-contaminated reads were mapped to *C. parvum* IOWA reference genome (http://cryptodb.org/common/downloads/release-35/CparvumIowaII/). For that, Bowtie2 v2.2.6 has been used, and only non-ambiguous alignments were selected. This means that we kept only uniquely mapped reads for DID and TUM1 Ion Torrent reads, and only reads that aligned concordantly exactly one time and with maximum alignment quality for paired-end Illumina CHR reads (‘-f 0×2’ and ‘-q 42’ in BAM output). Doing that, the number of incorrectly mapped reads that could be misleading for the subsequent variant calling step was minimized. Bedtools v2.25.0 coverage tool^61^, that computes both the depth and breadth of coverage, was used to obtain breadth of reference genome coverage. Variant calling was performed on the SAM files with the BCFtools commands from the SAMtools suite^62^. Only SNVs and indels with a Phred quality score above 30 and located in CDSs were selected. Intersection with CDSs was computed with BEDtools^61^ with the *C. parvum* IOWA GFF file. For DID and TUM1, indels falling in homopolymer regions (more than three identical nucleotides) were discarded, since they are likely to be artefactual sequencing errors. The variants obtained in VCF format were annotated with the SNPeff tool (http://snpeff.sourceforge.net/index.html) and manipulated with the extractField function of the SnpSift tool (http://snpeff.sourceforge.net/SnpSift.html) in order to easily display the effects and impacts of each variant.

### Analysis of shared CDSs variants in TUM1, DID and CHR isolates found by both MICRA and custom pipeline approaches

In order to uncover the genetic basis of the phenotypically observed differential virulence between *C. parvum* IOWA and the three more virulent DID, TUM1 and CHR isolates, SNV or indel-level common differences in CDSs between these three isolates have been identified. For each pipeline, a file containing all variants found between DID, TUM1 and CHR, in comparison with *C. parvum* IOWA was generated, as well as a file containing common CDSs and variants between the three more virulent isolates. Then, a comparison of the lists of covered CDSs and variants between DID, TUM1 and CHR isolates found either with MICRA or with the custom pipeline allowed us to identify shared CDSs and variants between the three isolates and identified by both pipelines. To note, a variant was considered common to the three isolates if it is present at exactly the same position and with the same variation. A SNV of interest was thus defined as a polymorphic site in *C. parvum* CDS that showed one nucleotide pattern for IOWA genome and another nucleotide pattern identical in DID, TUM1 and CHR sequences.

The SNVs of interest were classified as either non-synonymous or synonymous, SNVs eliminating start codon, causing premature termination codon or eliminating termination codon. PROVEAN software was also used to determine if non-synonymous variants are predicted to be functionally important^63^. Then, the set of SNV-associated genes (proteins) was evaluated for a variety of functional characteristics. Bio-informatic analyses of gene ontology (GO) and Kyoto Encyclopedia of Genes and Genomes (KEGG) pathway for their target genes were conducted with Blast2GO 4.1.9 program^64^. SNV-associated genes without GO terms were further evaluated using blastx and blastp homology searches from NCBI’s RefSeq database. Functional domains were predicted using SMART http://smart.embl-heidelberg.de/ and pfam http://pfam.xfam.org/^65,66^. The presence of signal peptides and trans-membrane (TM) domains was inferred using the SignalP program V4.1 (http://www.cbs.dtu.dk/services/SignalP/)^67^. GPIsom (http://gpi.unibe.ch/) was used to detect GPI-anchored proteins (both C- and N-terminal signal sequences)^68^. SNV-associated genes of interest were also assessed for their identity with the already described putative *Cryptosporidium* virulence factors; ^11,15^(ProtVirDB database (http://bioinfo.icgeb.res.in/protvirdb/)). The expression profiles of the variants proteins during the *Cryptosporidium* life-cycle were collected at CryptoDB (https://cryptodb.org/) in the Transcriptomics section according to RNAseq datasets provided by Giovanni Widmer Christoph Lippuner.

Finally, distribution of SNVs in the 3 *C. parvum* genomes sequenced in this study in comparison with the published reference IOWA genome was drawn for the 8 chromosomes with BedTools makewindows, coverage and counts options in R, and circos v0.69 (http://circos.ca/)^69^ for graphs.

### **SNVs confirmation by Sanger sequencing and description of novel SNVs in the*C. parvum*genome**

Sanger sequencing was used to validate SNVs identified by Ion-Torrent sequencing in 8 randomly selected SNV-associated genes. CDSs of interest, sequences of primers used to amplify DNA fragments (before WGA) as well as PCR conditions are listed in Supplementary Table S6. After validation of amplified products (5 µL) by agarose gel electrophoresis, PCR products were purified and sequenced directly on both strands, using the forward and reverse PCR primers, by Genoscreen (Lille, France). Obtained sequences were analyzed using the BioEdit v7.0.1 package, and compared with the sequences obtained from Ion-Torrent sequencing by the ClustalW Multiple sequence alignment tool. Moreover, all SNVs of interest (*i.e*. common SNVs found in DID, TUM1 and CHR isolates) were searched on CryptoDB (http://cryptodb.org/) to identify whether these SNVs had already been described in the different isolates of *C. parvum* available in the CryptoDB database.

## Supplementary information

Supplementary Files.

Suppl. Table S1.

Suppl. Table S2.

Suppl. Table S3.

Suppl. Table S4.

Suppl. Table S5.

Suppl. Table S6.
