# Supplementary material for: Genetic basis for virulence differences of various Cryptosporidium parvum carcinogenic isolates
Source: Sci Rep. 2020 Apr 30;10:7316. doi: 10.1038/s41598-020-64370-0 (PMC7193590; doi:10.1038/s41598-020-64370-0)
Supplement: Supplementary file 1 — Supplementary Files. [file 41598_2020_64370_MOESM1_ESM.pdf]

Genetic basis for virulence differences of various *Cryptosporidium parvum* carcinogenic isolates

Christophe Audebert[1,2], Franck Bonardi[3], Ségolène Caboche[2,4], Karine Guyot[4], Hélène Touzet[3,5], Sophie Merlin[1,2], Nausicaa Gantois[4], Colette Creusy[6], Dionigia Meloni[4], Anthony Mouray[7], Eric Viscogliosi[4], Gabriela Certad[4,8], Sadia Benamrouz-Vanneste[4,9], Magali Chabé[4*]

[1]Gènes Diffusion, 3595, route de Tournai, 59501 Douai, France ; [2]PEGASE-Biosciences, Institut Pasteur de Lille, Lille, France ; [3]Bilille, Institut Pasteur de Lille, Lille, France ; [4]Univ. Lille, CNRS, Inserm, CHU Lille, Institut Pasteur de Lille, U1019 – UMR 8204 – CIIL – Centre d'Infection et d'Immunité de Lille, Lille, France ; [5]CNRS, Univ. Lille, Inria, UMR 9189 - CRIStAL - Centre de Recherche en Informatique Signal et Automatique de Lille, Lille, France ; [6]Service d'Anatomie et de Cytologie Pathologiques, Groupement des Hôpitaux de l'Institut Catholique de Lille (GHICL), Lille, France ; [7]Plateforme d'Expérimentations et de Hautes Technologies Animales, Institut Pasteur de Lille, Lille, France ; [8]Délégation à la Recherche Clinique et à l'Innovation, Groupement des Hôpitaux de l'Institut Catholique de Lille, Lille, France ; [9]Equipe Ecologie et biodiversité, Unité de Recherche Smart and Sustainable Cities, Faculté de Gestion Economie et Sciences, Institut Catholique de Lille, France

*Corresponding author

E-mail: magali.chabe@univ-lille.fr

Suppl. File 1. Identification of contaminant bacterial reads of WGS data of DID, TUM1 and CHR strains with MICRA:

MICRA was first run in automatic way using bacterial databases. The pre-process module allows to identify close reference genomes and plasmids blasting a subset of reads (1,000 reads for genomes and 10,000 reads for plasmids) against the reference sequences (see the MICRA publication for more details about parameters). The top five genomes and top five or ten plasmids for DID, TUM1 and CHR strains returned by the pre-process step are given below and the number of read matches are shown.

DID strain

Genomes

Escherichia-coli-ED1a (NC_011745.1) - **3 read matches**
Pseudomonas-putida-W619 (NC_010501.1) - **1 read match**
Propionibacterium-acnes-SK137 (NC_014039.1) - **1 read match**
Pseudomonas-brassicacearum-subsp.-brassicacearum-NFM421 (NC_015379.1) - **1 read match**
Propionibacterium-acnes-KPA171202 (NC_006085.1) - **1 read match**

Plasmids

Corynebacterium-renale (X99132.2) - **87 read matches**
Escherichia-coli (HQ328804.1) - **43 read matches**
Escherichia-coli-ABU-83972 (CP001833.1) - **33 read matches**
Escherichia-coli (JF436966.1) - **13 read matches**
Shigella-flexneri (JF813186.1) - **4 read matches**
Escherichia-coli (EU999782.1) - **1 read match**
Corynebacterium-diphtheriae (AY061891.1) - **1 read match**
Corynebacterium-diphtheriaegi: (AF492560.1) - **1 read match**
Ralstonia-solanacearum-CMR15 (FP885896.1) - **1 read match**
Enterococcus-faecium (EU327398.1) - **1 read match**

Any close reference genome was identified by MICRA, the closest one showing only a coverage of 0.18%. This low percentage of coverage showed that no bacterial contamination was observed for the DID strain.

TUM1 strain

Genomes

Lactobacillus-reuteri-JCM-111 (NC_010609.1) - **49 read matches**
Lactobacillus-reuteri-DSM-20016 (NC_009513.1) - **47 read matches**
Lactobacillus-reuteri-SD2112 (NC_015697.1) - **46 read matches**
Enterococcus-faecalis-V583 (NC_004668.1) - **7 read matches**
Lactobacillus-johnsonii-FI9785 (NC_013504.1) - **5 read matches**

Plasmids

Lactobacillus-reuteri (HQ015473.1) - **23 read matches**
Lactobacillus-sanfranciscensis-TMW-1.1304 (CP002462.1) - **8 read matches**
Lactobacillus-reuteri-SD2112 (CP002846.1) - **3 read matches**
Bacillus-megaterium-WSH-002 (CP003018.1) - **3 read matches**
Enterococcus-faecalis-V583 (AE016831.1) - **1 read match**
Lactobacillus-reuteri-SD2112 (CP002845.1) - **1 read match**
Azospirillum-sp.-B510 (AP010951.1) - **1 read match**
Lactococcus-lactis-subsp.-lactis-K214 (X92946.1) - **1 read match**
Methylobacterium-radiotolerans-JCM-2831 (CP001002.1) - **1 read match**
Enterococcus-faecalis (AB374546.1) - **1 read match**

The closest reference genome was *Lactobacillus reuteri DSM 20016* (NC_009513.1) covered at 78% and showing a bacterial contamination in TUM1 data. No plasmid was identified in this step.

CHR strain

Genomes

Nocardia-farcinica-IFM-10152-DNA (NC_006361.1) - **137 read matches**
Rhodococcus-jostii-RHA1 (NC_008268.1) - **47 read match**
Rhodococcus-opacus-B4-DNA (NC_012522.1) - **46 read match**
Rhodococcus-equi-103S (NC_014659.1) - **44 read match**
Mycobacterium-sp.-MCS (NC_008146.1) - **33 read match**

Plasmids

Streptomyces-cattleya-DSM-46488 (CP003229.1) - **21 read matches**
Rhodococcus-jostii-RHA1 (CP000432.1) - **18 read matches**
Mycobacterium-chubuense-NBB4 (CP003054.1) - **13 read matches**
Rhodococcus-opacus-B4 (AP011116.1) - **10 read matches**
Streptomyces-rochei (AB088224.2) - **9 read matches**

Any close reference genome was identified by MICRA, the closest one showing only a coverage of 4.42%. This low percentage of coverage showed that no bacterial contamination was observed for the CHR strain. No plasmid was identified in this step.

Suppl. File 2. Identification of contaminant bacterial reads of WGS data of DID, TUM1 and CHR strains with Kaiju:

By twisting the normal use of Kaiju (Menzel *et al*., 2016), which is a program for sensitive taxonomic classification of high-throughput sequencing reads from metagenomic WGS or metatranscriptomics experiments, each sequencing read can be assigned to a taxon in the NCBI taxonomy. By using protein-level classification, Kaiju achieves a higher sensitivity compared with methods based on nucleotide comparison. Thus, Kaiju helps to identify by which species the reads could have been contaminated.

After quality check and trimming step, TUM1 reads were imported and displayed using the Krona radial space filling. Taxonomy nodes are shown as nested sectors arranged from the top level of the hierarchy at the center and progressing outward. The chart is zoomed at the root to show only classified reads, here 31% of reads were unclassified (not shown). Of the classified reads, 28% were assigned to the Bacterial domain, of which 25% only belongs to the genus *Lactobacillus*. To note, no bacterial contamination was found with Kaiju for DID and CHR strains.

a. TUM1's reads assignments distribution. Of these reads, 31% are unclassified and are excluded from this visualization

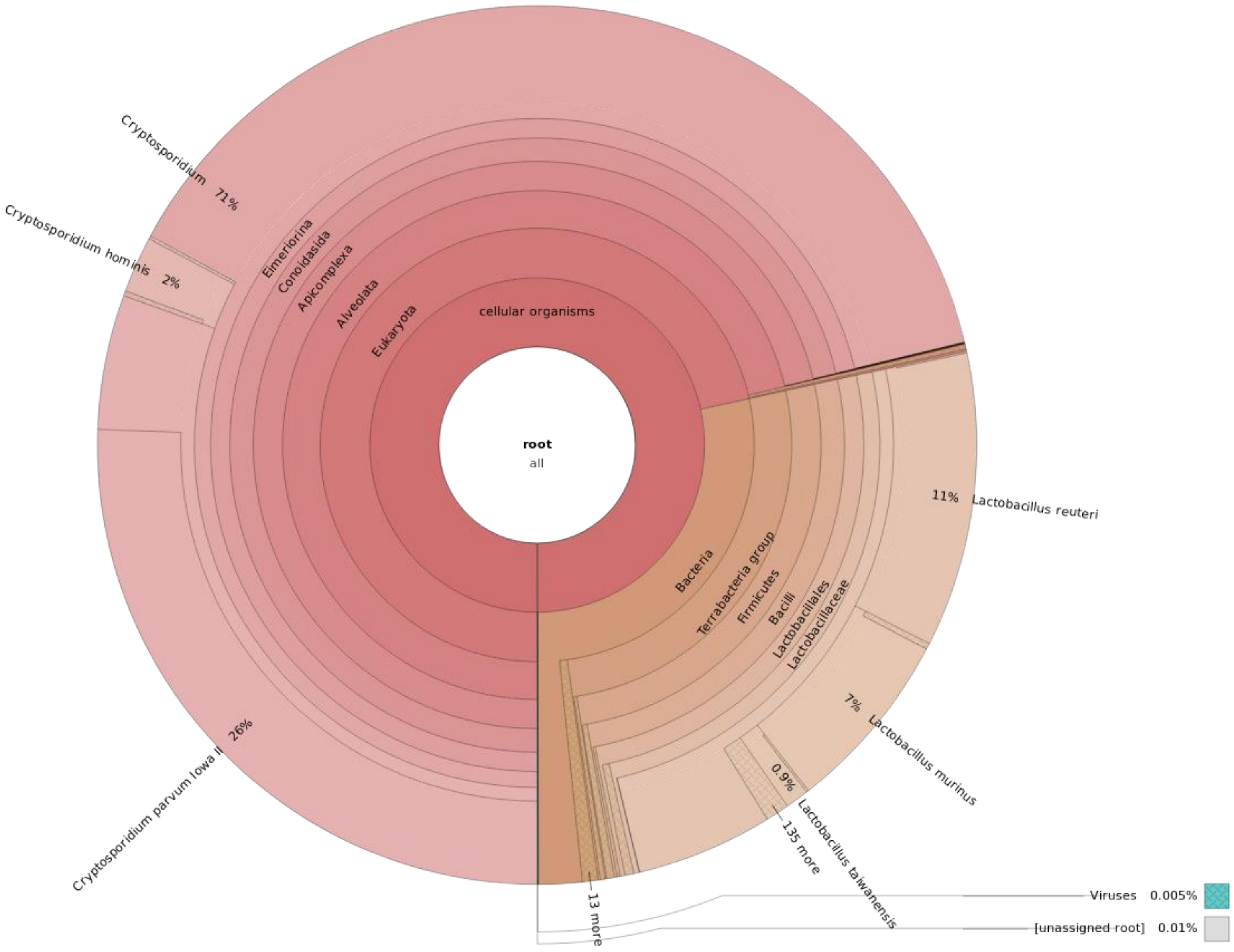

b. Zoom of the bacterial domain distribution of TUM1 reads

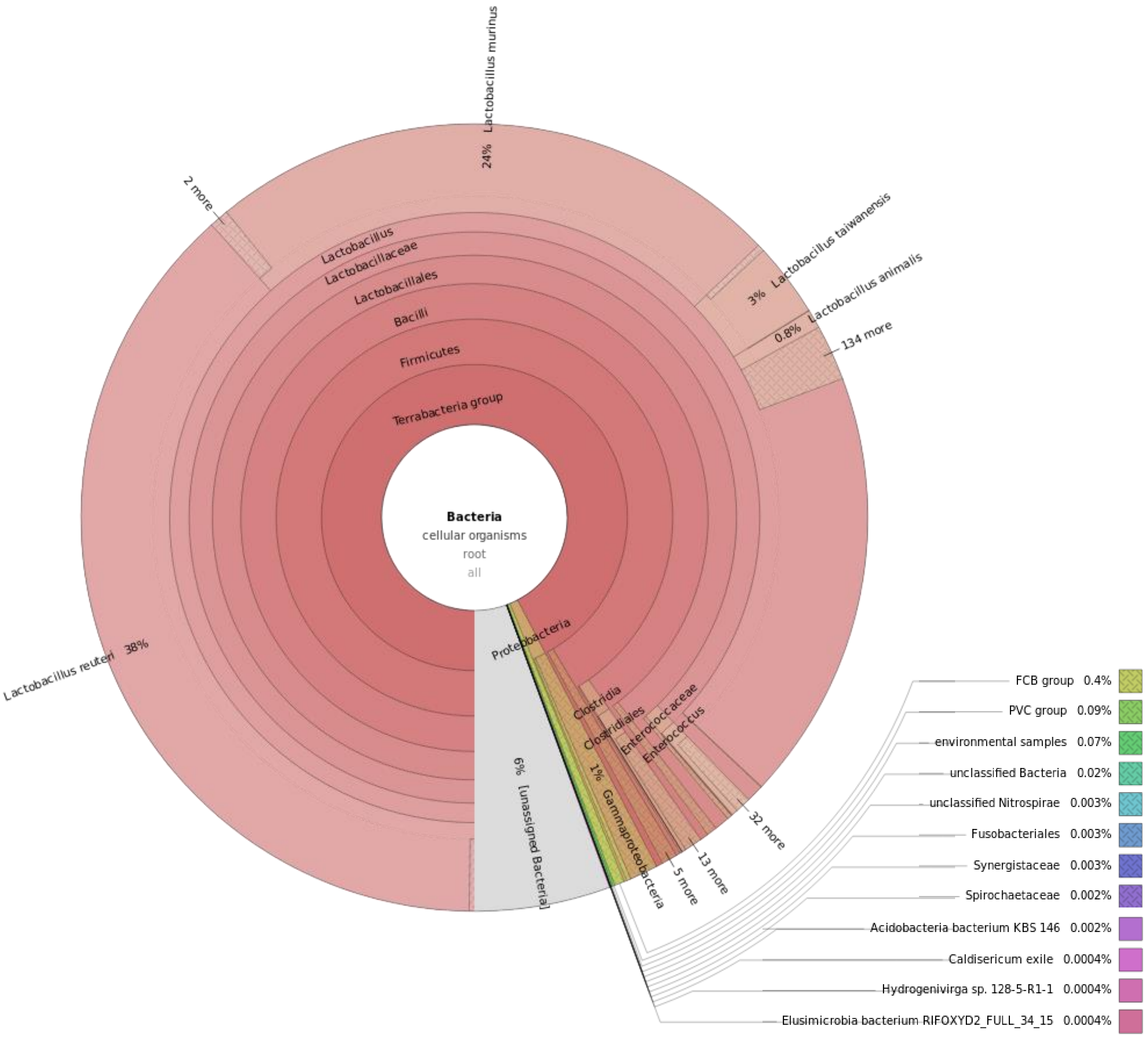

Using the reference genomes of the bacterial species assigned by Kaiju, thousand reads from TUM1 belonging to these contaminating species were identified. Below is a list of the main contaminants (on the left the number of reads assigned and on the right the species concerned). Species with less than 400 reads are not shown here.

205106 *Lactobacillus reuteri*
145178 *Lactobacillus murinus*
20659 *Lactobacillus taiwanensis*
4800 *Lactobacillus animalis*
3934 *Enterococcus faecalis*
3533 *Lactobacillus murinus* ASF361
3324 *Lactobacillus apodemi*
2271 *Lactobacillus johnsonii*
1768 *Lactobacillus taiwanensis* DSM 21401
1606 *Lactobacillus gasseri*
1149 *Enterorhabdus caecimuris*
1100 *Lactobacillus salivarius*
1051 *Staphylococcus epidermidis*
1007 *Lachnospiraceae bacterium* A4
617 *Bacillus cereus* VD133
595 *Escherichia coli*
559 *Pseudomonas* sp. ok266
425 *Acetobacter malorum*

492,686 reads identified as bacterial in the raw data were discarded, corresponding to 88.04% of reads assigned as bacterial by Kaiju. Thus, it remained 1,063,669 reads (cleaned, trimmed and "decontaminated") including 64,756 bacterial reads that alignment tools have failed to align with their reference bacterial genome but which were not aligned with the genome of *C. parvum* IOWA II.

Suppl. File 3. Distribution of SNVs in *C. parvum* DID (a), TUM1 (b) and CHR (c) strain genomes in comparison with the reference IOWA II genome. Circles from outer to inner represents: numbered chromosomes, each chromosome has a color and there is a small scale for each chromosome that represents the total length of the chromosome; reads coverage rate per 2500 bp window; and number of variants by these 2500 bp windows. The last two were both calculated on R package with various BedTools options ('makewindows', 'coverage' and 'counts'). Graphs were made with circos v0.69 (http://circos.ca/) Krzywinski et al. (Krzywinski *et al.*, 2009).

a.

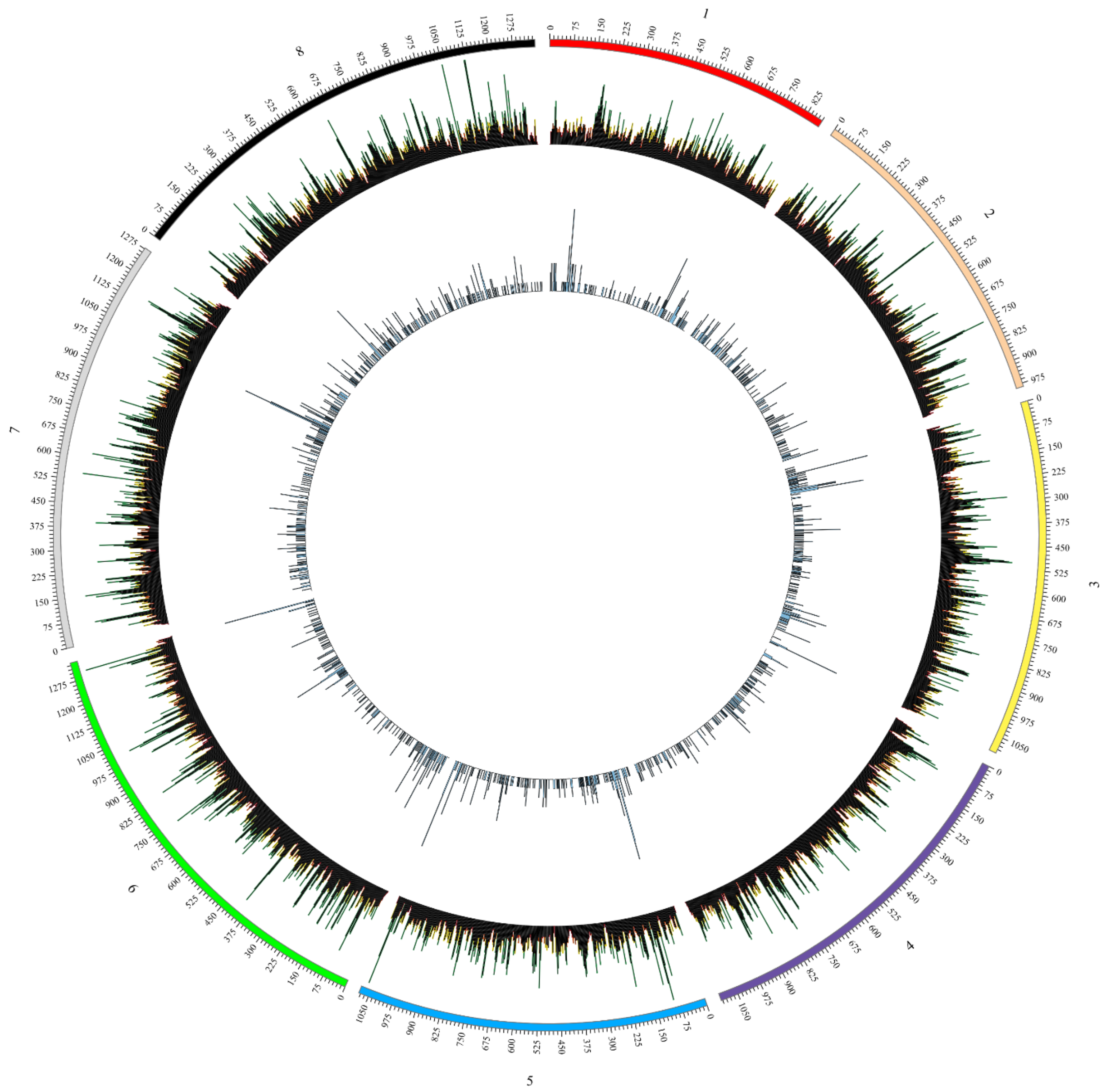

b.

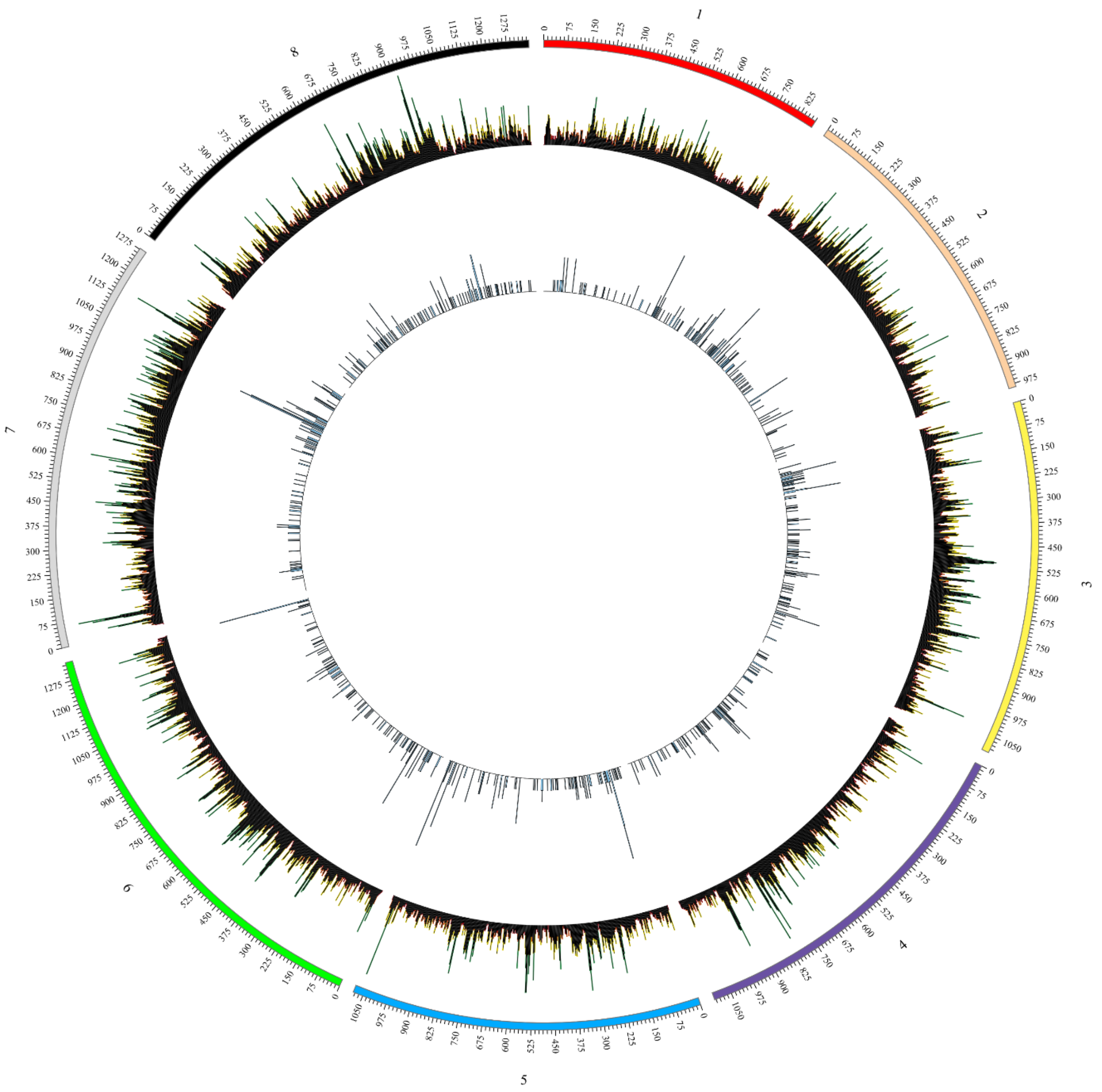

c.

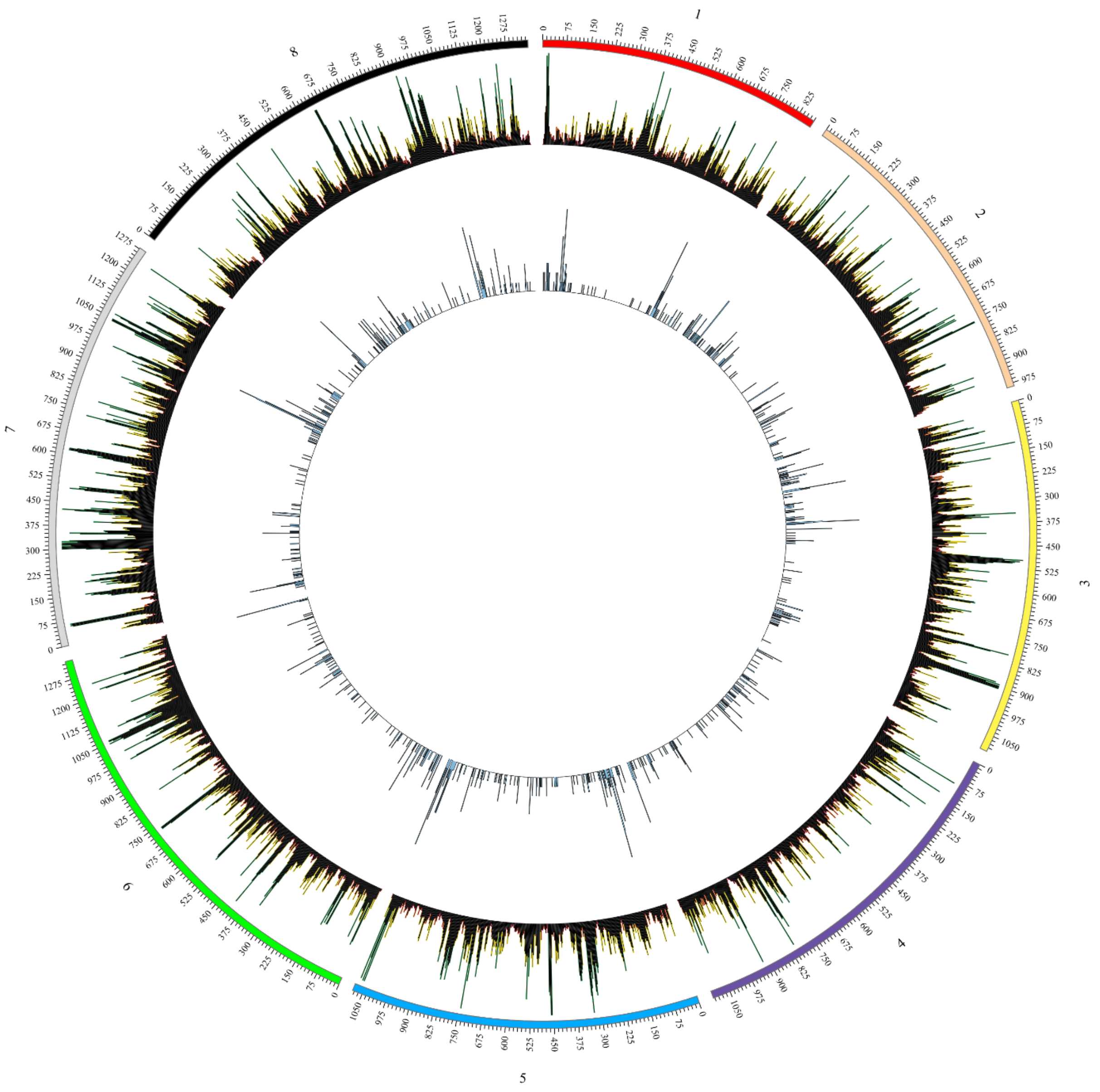

Suppl. File 4. Blast2GO analysis of 90 CDSs of interest (encompassing common SNPs found between DID, TUM1 and CHR strains). Combined graphs were performed in Blast2GO at level 3 and 4 for Molecular Function, Cellular Component and Biological Process aspects of Gene Ontology. Values within parentheses are the number of sequences / % associated with each Gene Ontology term.

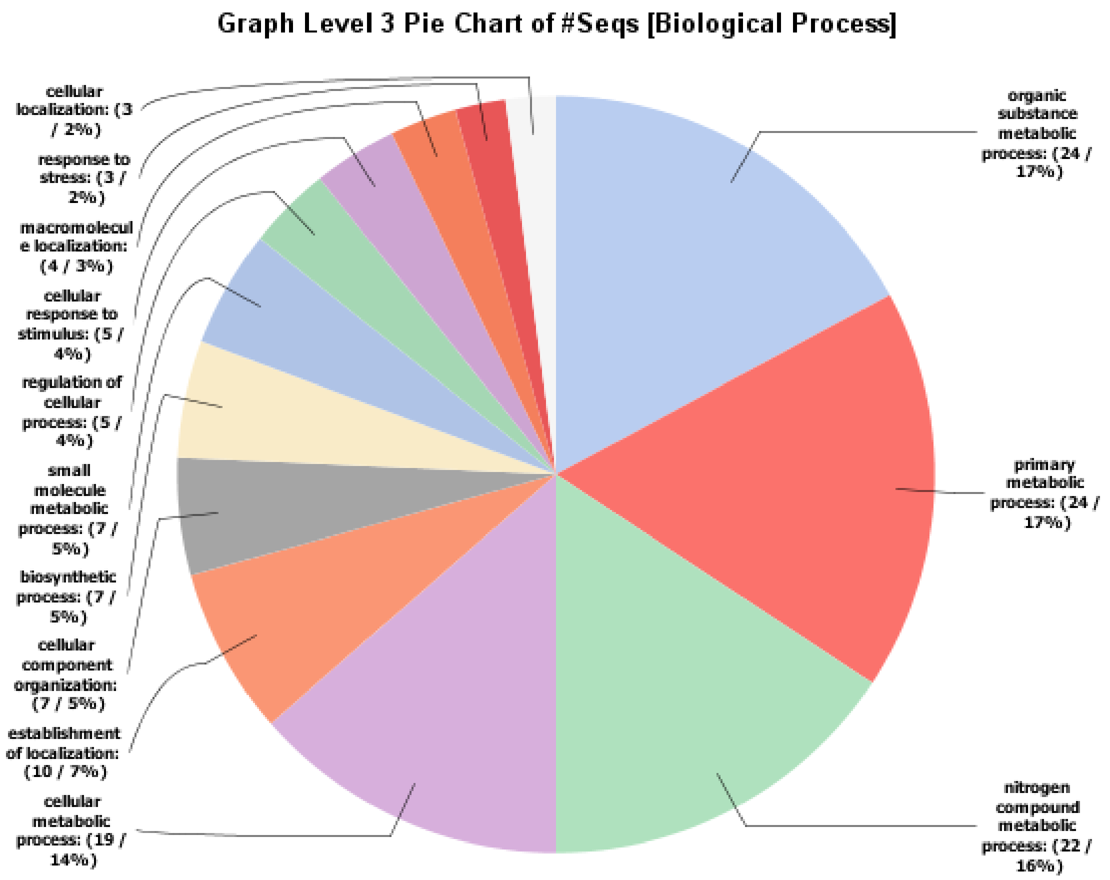

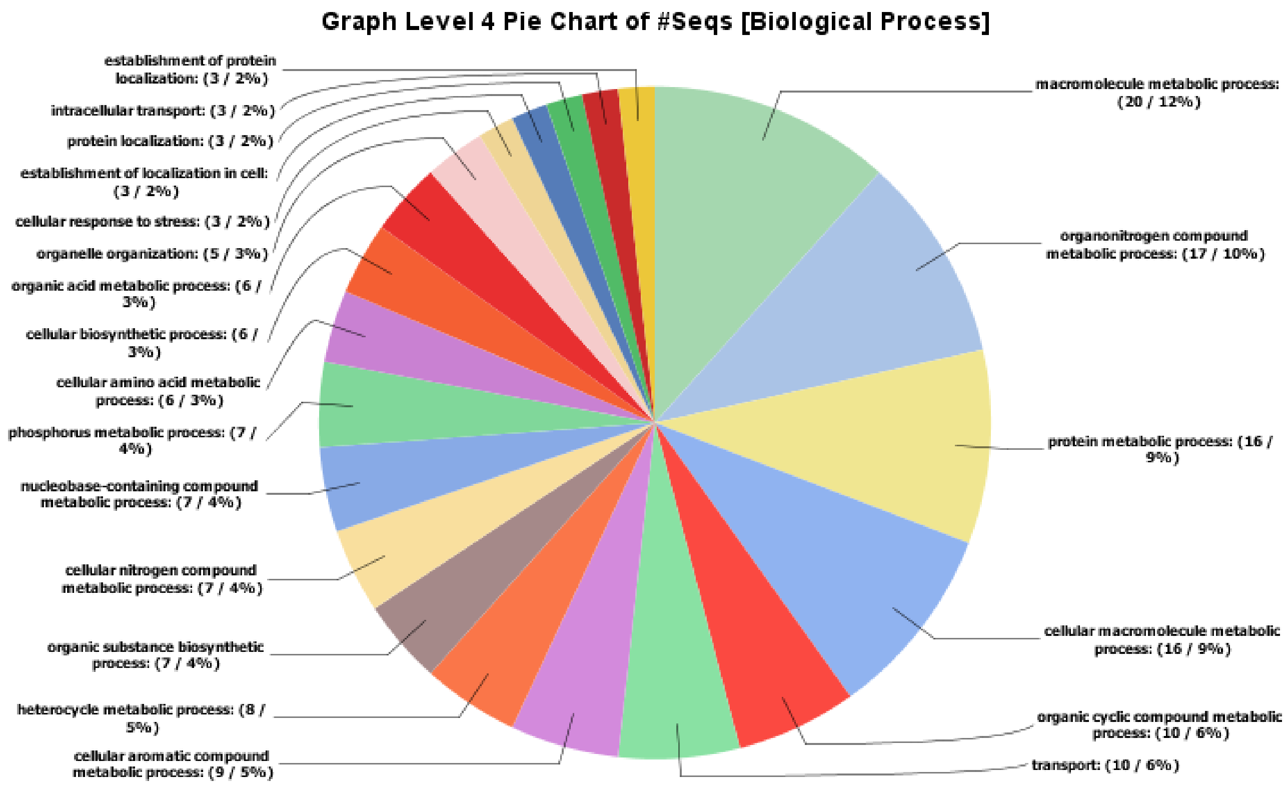
Graph Level 4 Pie Chart of #Seqs [Biological Process]
establishment of protein localization: (3 / 2%)
intracellular transport: (3 / 2%)
protein localization: (3 / 2%)
establishment of localization in cell: (3 / 2%)
cellular response to stress: (3 / 2%)
organelle organization: (5 / 3%)
organic acid metabolic process: (6 / 3%)
cellular biosynthetic process: (6 / 3%)
cellular amino acid metabolic process: (6 / 3%)
phosphorus metabolic process: (7 / 4%)
nucleobase-containing compound metabolic process: (7 / 4%)
cellular nitrogen compound metabolic process: (7 / 4%)
organic substance biosynthetic process: (7 / 4%)
heterocycle metabolic process: (8 / 5%)
cellular aromatic compound metabolic process: (9 / 5%)
macromolecule metabolic process: (20 / 12%)
organonitrogen compound metabolic process: (17 / 10%)
protein metabolic process: (16 / 9%)
cellular macromolecule metabolic process: (16 / 9%)
organic cyclic compound metabolic process: (10 / 6%)
transport: (10 / 6%)

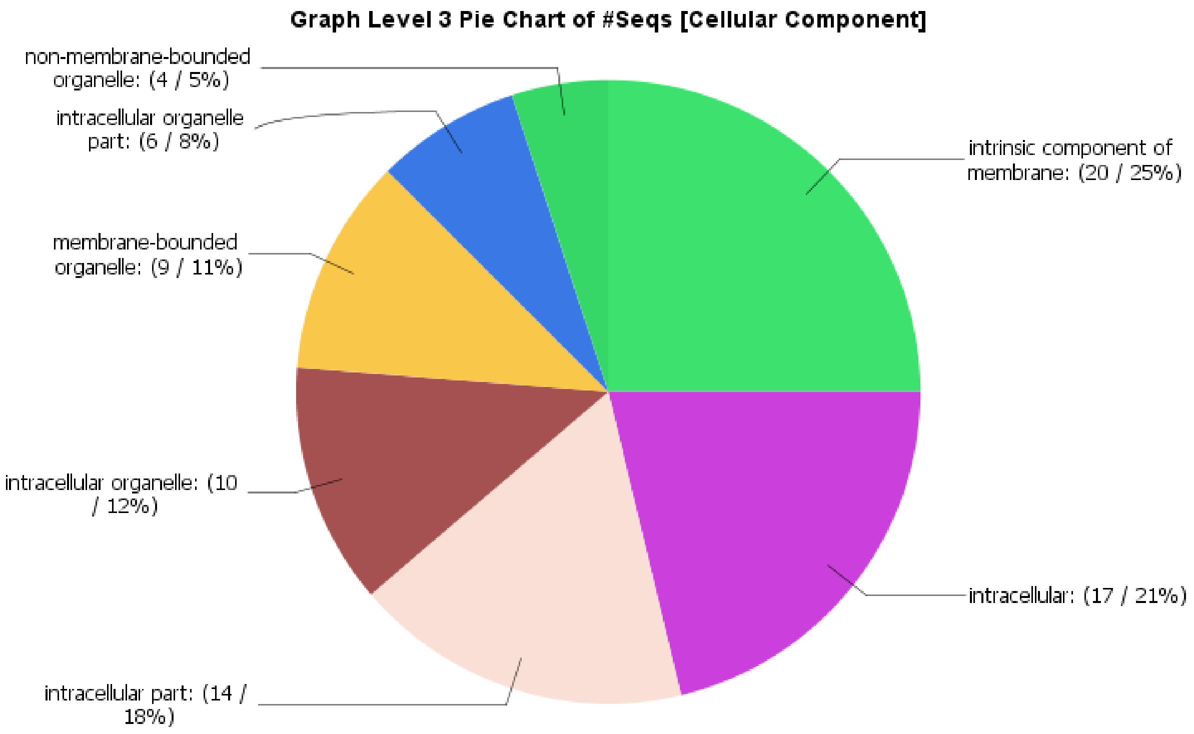
Graph Level 3 Pie Chart of #Seqs [Cellular Component]
non-membrane-bounded organelle: (4 / 5%)
intracellular organelle part: (6 / 8%)
membrane-bounded organelle: (9 / 11%)
intracellular organelle: (10 / 12%)
intracellular part: (14 / 18%)
intrinsic component of membrane: (20 / 25%)
intracellular: (17 / 21%)

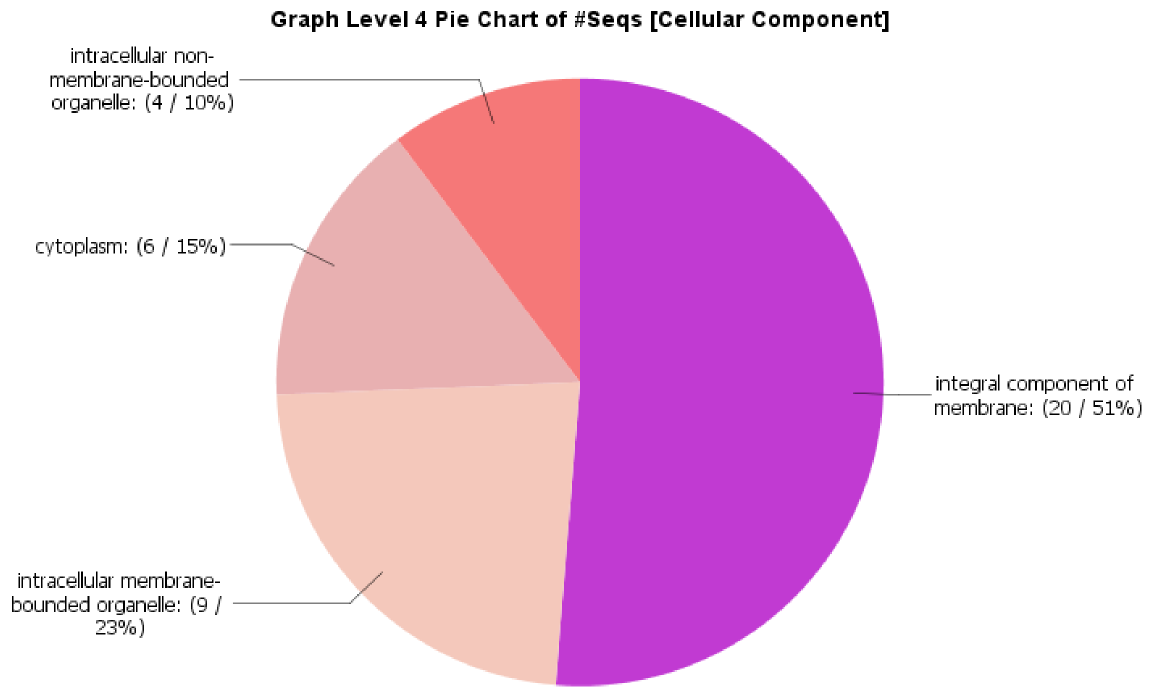
Graph Level 4 Pie Chart of #Seqs [Cellular Component]
intracellular non-membrane-bounded organelle: (4 / 10%)
cytoplasm: (6 / 15%)
integral component of membrane: (20 / 51%)
intracellular membrane-bounded organelle: (9 / 23%)

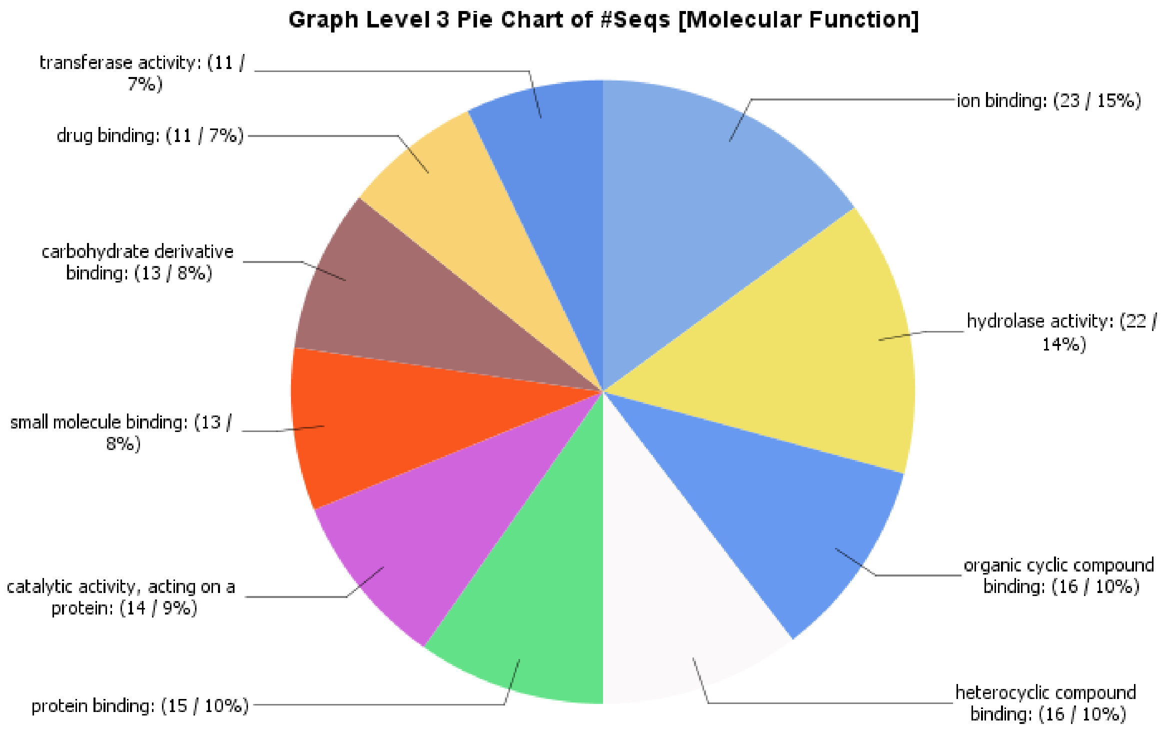
Graph Level 3 Pie Chart of #Seqs [Molecular Function]
transferase activity: (11 / 7%)
ion binding: (23 / 15%)
drug binding: (11 / 7%)
carbohydrate derivative binding: (13 / 8%)
hydrolase activity: (22 / 14%)
small molecule binding: (13 / 8%)
organic cyclic compound binding: (16 / 10%)
catalytic activity, acting on a protein: (14 / 9%)
protein binding: (15 / 10%)
heterocyclic compound binding: (16 / 10%)

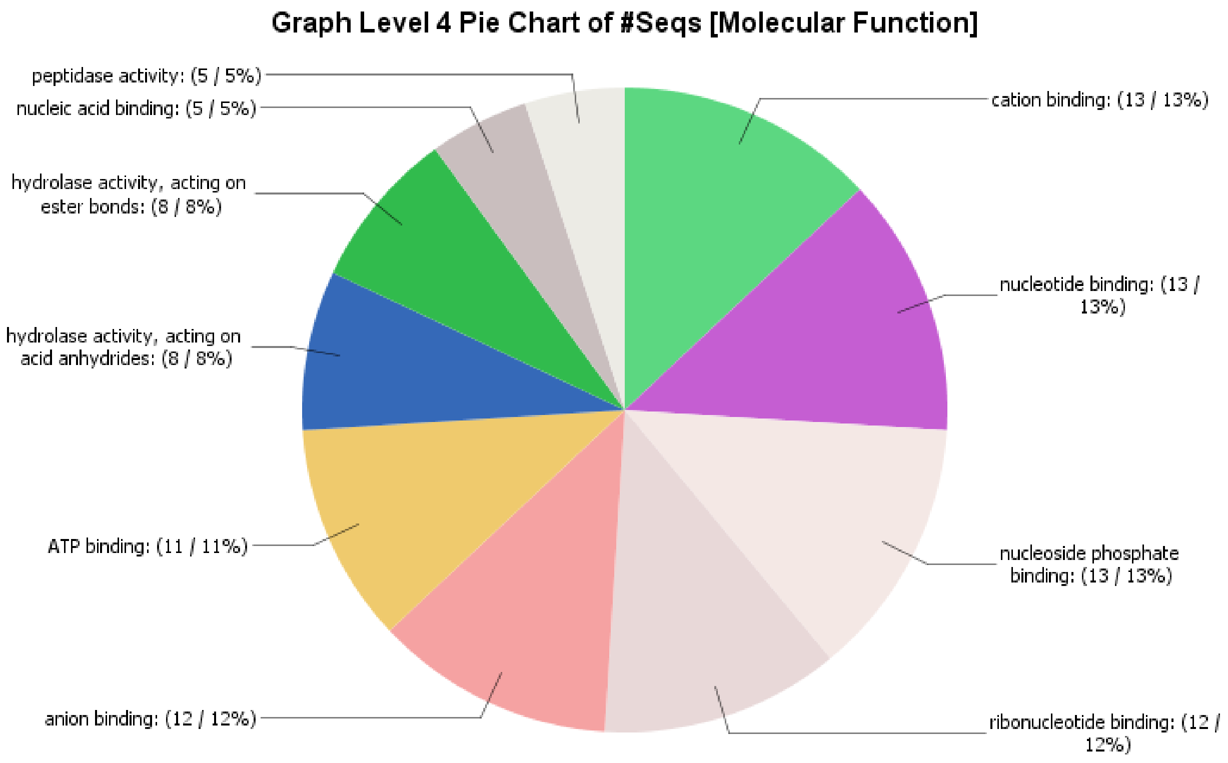
Graph Level 4 Pie Chart of #Seqs [Molecular Function]
peptidase activity: (5 / 5%)
nucleic acid binding: (5 / 5%)
hydrolase activity, acting on ester bonds: (8 / 8%)
hydrolase activity, acting on acid anhydrides: (8 / 8%)
ATP binding: (11 / 11%)
anion binding: (12 / 12%)
cation binding: (13 / 13%)
nucleotide binding: (13 / 13%)
nucleoside phosphate binding: (13 / 13%)
ribonucleotide binding: (12 / 12%)
